# Microcomputed tomography versus plethysmometer and electronic caliper in the measurements of lymphedema in the hindlimb of mice

**DOI:** 10.1038/s41598-022-16311-2

**Published:** 2022-07-18

**Authors:** Amar Bucan, Alexander Wiinholt, Farima Dalaei, Oke Gerke, Christian Rønn Hansen, Jens Ahm Sørensen

**Affiliations:** 1 Research Unit for Plastic Surgery, Odense University Hospital, University of Southern Denmark, Odense, Denmark; 2 Department of Nuclear Medicine, Odense University Hospital, Odense, Denmark; 3 Department of Clinical Research, University of Southern Denmark, Odense, Denmark; 4 Laboratory of Radiation Physics, Odense University Hospital, Odense, Denmark

**Keywords:** Breast cancer, Preclinical research

## Abstract

Lymphedema affects 20% of women diagnosed with breast cancer. It is a pathology with no known cure. Animal models are essential to explore possible treatments to understand and potentially cure lymphedema. The rodent hindlimb lymphedema model is one of the most widely used. Different modalities have been used to measure lymphedema in the hindlimb of mice, and these are generally poorly assessed in terms of the interrater agreement; thus, there could be a risk of measuring bias and poor reproducibility. We examined the interrater agreement of µCT-scans, electronic caliper thickness of the paw and plethysmometer in the measurement of lymphedema in the hindlimb of mice. Three independent raters assessed 24 C57BL6 mice using these three modalities four times (week 1, 2, 4 and 8) with a total of 96 samples. The mean interrater differences were then calculated. The interrater agreement was highest in the µCT-scans, with an extremely low risk of measurement bias. The interrater agreement in the plethysmometer and electronic caliper was comparable with a low to moderate risk of measurement bias. The µCT-scanner should be used whenever possible. The electronic caliper should only be used if there is no µCT-scanner available. The plethysmometer should not be used in rodents of this size.

## Introduction

Lymphedema remains a pathology with no cure^1^. Approximately 20% of women diagnosed with breast cancer will develop lymphedema due to treatments including mastectomy, axillary lymph node dissection and/or radiotherapy^2^. The primary therapy is conservative care consisting of compression, manual lymph drainage and skincare^3^. Secondary therapy is used when patients experience inadequate results of the primary therapy. It consists of microsurgical treatment with varying success rates^4^. Animal models are being used to explore possible treatments to better understand and potentially cure lymphedema. The recurring problem is that animal research lacks standardized parameters to measure lymphedema objectively^5^.

Animal models have been used since 1968 in lymphedema research^6^. Different animals have been used throughout years. In 2017, 80% of all published models were rodent^6^. The rodent tail and hindlimb models are most commonly used^6^. The hindlimb model is considered to be the most eligible, being an easily accessible, cost-effective and reliable lymphedema model^7^.

When measuring the lymphedema in the hindlimb of mice, different modalities have been used and these are generally poorly understood^5,8^. Most of these modalities have yet to be properly assessed in terms of interrater agreement^9^.

Conventional measuring techniques used as surrogate parameters for hindlimb lymphedema consist of paw thickness using an electronic caliper, circumferential length of the hindlimb and planimetric analysis using a photograph^5^. A recent study examined these conventional techniques and found the electronic caliper to have a high interrater agreement and the fewest outliers compared to the other two techniques^5^. Water displacement technique (plethysmometer) is often used in rodent hindlimb lymphedema research^10–12^. However, the interrater agreement has never been examined.

In recent years, 3D hindlimb volumetry such as micro-computed tomography (µCT), magnetic resonance imaging (MRI) and high-resolution ultrasound (hrUS) have been introduced for rodent hindlimb volumetry^5,13–15^. In 2016, Frueh et al.^5^ examined these three modalities finding high interrater agreement among all three but with µCT as the modality with the lowest risk of measurement bias.

Two studies have investigated interrater agreement of µCT-scans and shown an extremely low risk of measuring bias^5,16^. To our knowledge, electronic caliper measurements have only been examined in a single study in terms of interrater agreement^5^, and the plethysmometer has never been examined. Thus, lymphedema studies on the hindlimbs of mice are being conducted without proper knowledge of possible measuring bias and reproducibility, and further research is needed to standardize parameters for measuring lymphedema in the hindlimb of mice.

The primary aim of this study was to examine the interrater agreement of µCT-scans, electronic caliper thickness of the paw and plethysmometer in the measurement of lymphedema in the hindlimb of mice. The secondary aim was to conduct a correlation analysis of the µCT-scans with the electronic caliper and plethysmometer. The population of interest were C57BL/6 mice, and the rater population of interest consisted of three medical doctors. The Guidelines for Reporting Reliability and Agreement Studies (GRRAS)^17^ were applied.

## Results

The results are presented in separate paragraphs for measurements, interrater agreement and correlation analysis.

### Measurements

The µCT-scans were measured in cubic millimeters (mm^3^), the plethysmometer in milliliters (ml) and the electronic caliper in millimeters (mm).

#### µCT-scans

The mean volume across all mice for the lymphedema hindlimb was 220.1 mm^3^ and 153.7 mm^3^ for the control hindlimb.

**Figure 1:**
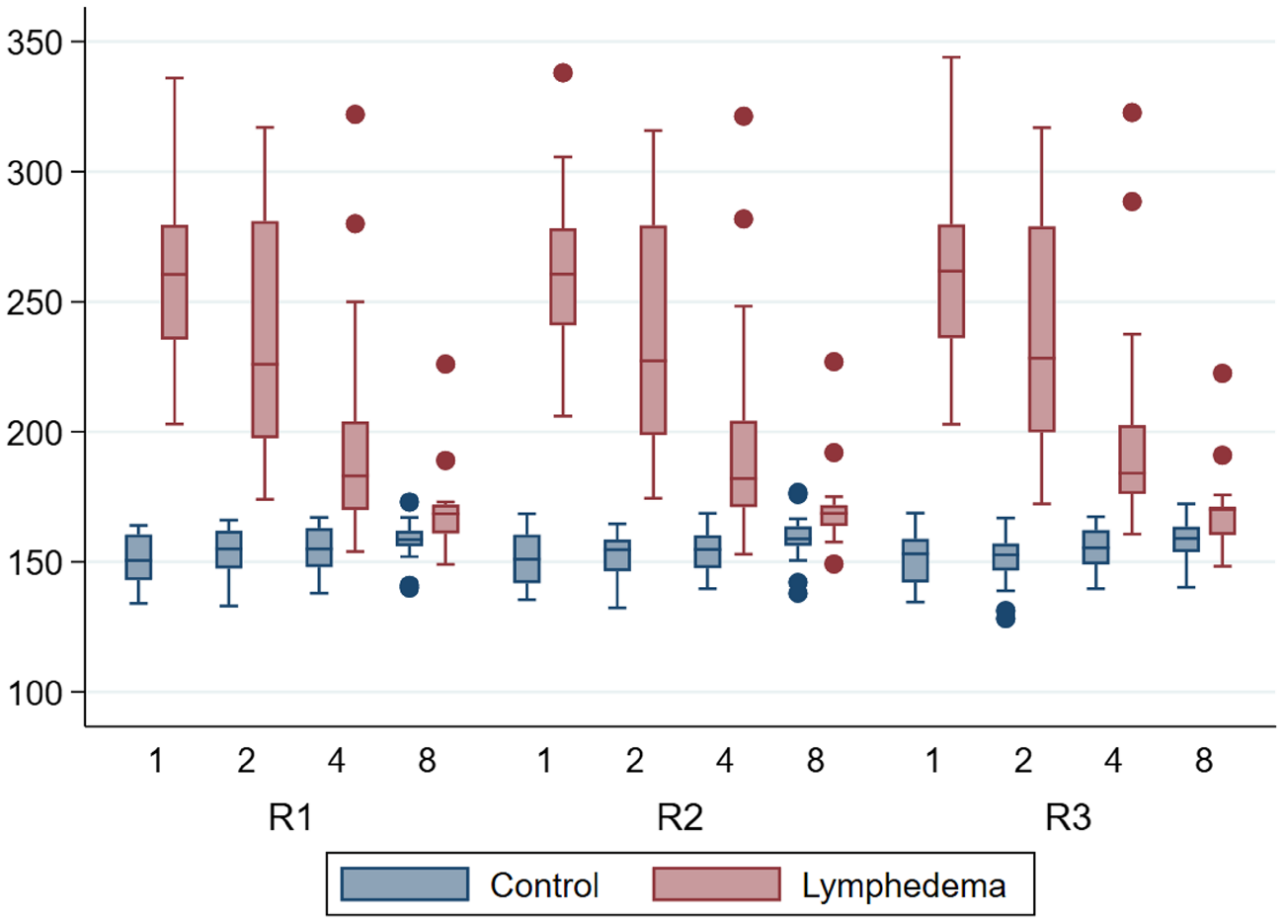
Descriptive boxplot showing the µCT-scans by rater, week and group. The y-axis is in cubic millimeters [mm^3^]. The x-axis is weeks. *CT* computed tomography, *μ* micro, *R1* rater 1, *R2* rater 2, *R3* rater 3.

The volumes of the mice assessed by µCT-scans varied throughout all 8 weeks from 149 mm^3^ to 336 mm^3^ (R1), 149.2 mm^3^ to 338 mm^3^ (R2) and 148.3 mm^3^ to 344 mm^3^ (R3) in the lymphedema hindlimb, and 133 mm^3^ to 173 mm^3^ (R1), 132.3 mm^3^ to 176.7 mm^3^ (R2) and 128.2 mm^3^ to 172.3 mm^3^ (R3) in the control hindlimb. The results of the µCT-scans are graphed in Fig. 1.

#### Plethysmometer

The mean volume across all mice for the lymphedema hindlimb was 0.09 ml, and 0.06 ml for the control hindlimb.

**Figure 2:**
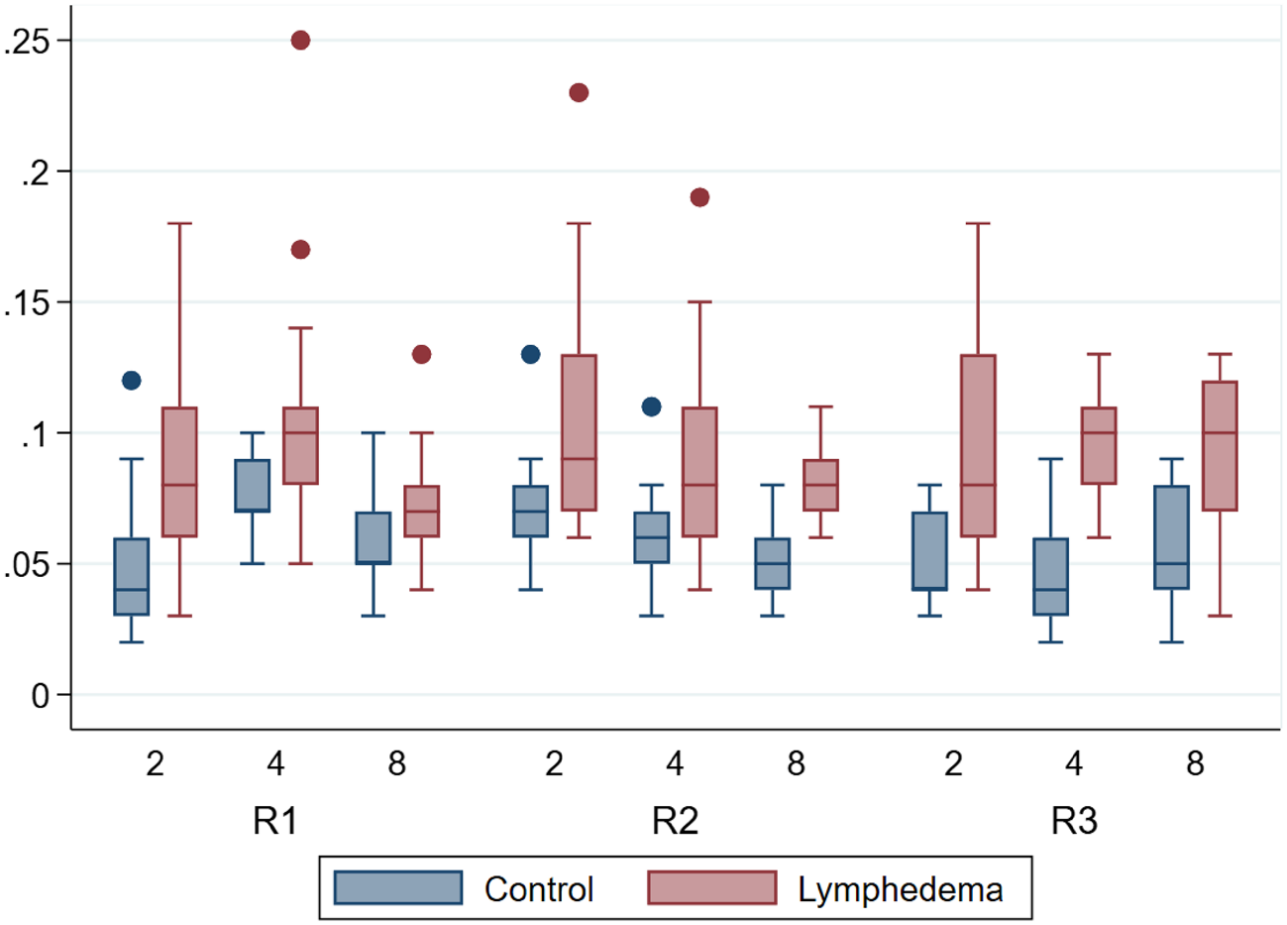
Descriptive boxplot showing plethysmometer by rater, week and group. The y-axis is in milliliters [mL]. The x-axis is weeks. *R1* rater 1, *R2* rater 2, *R3* rater 3.

The volumes of the mice assessed by plethysmometer varied from 0.03 ml to 0.25 ml (R1), 0.04 ml to 0.23 ml (R2) and 0.03 ml to 0.18 ml (R3) in the lymphedema hindlimb, and 0.02 ml to 0.12 ml (R1), 0.03 ml to 0.13 ml (R2) and 0.02 ml to 0.09 ml (R3) in the control hindlimb. The results of the plethysmometer measurements are graphed in Fig. 2.

#### Electronic caliper

The mean thickness of the paw across all mice for the lymphedema hindlimb was 3.27 mm, and 2.55 mm for the control hindlimb.

**Figure 3:**
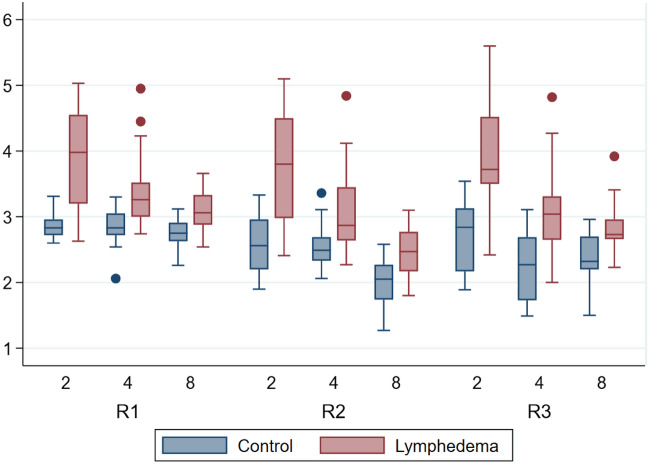
Descriptive boxplot showing electronic caliper [mm] by rater, week and group. The y-axis is in millimeters [mm]. The x-axis is weeks. *R1* rater 1, *R2* rater 2, *R3* rater 3.

The thickness of the paw of the mice assessed by electronic caliper varied from 2.54 mm to 5.03 mm (R1), 1.8 mm to 5.10 mm (R2) and 2 mm to 5.60 mm (R3) in the lymphedema hindlimb and 2.06 mm to 3.31 mm (R1), 1.27 mm to 3.36 mm (R2) and 1.49 mm to 3.54 mm (R3) in the control hindlimb. The results of the electronic caliper measurements are graphed in Fig. 3.

### Interrater agreement

#### µCT-scans

**Figure 4:**
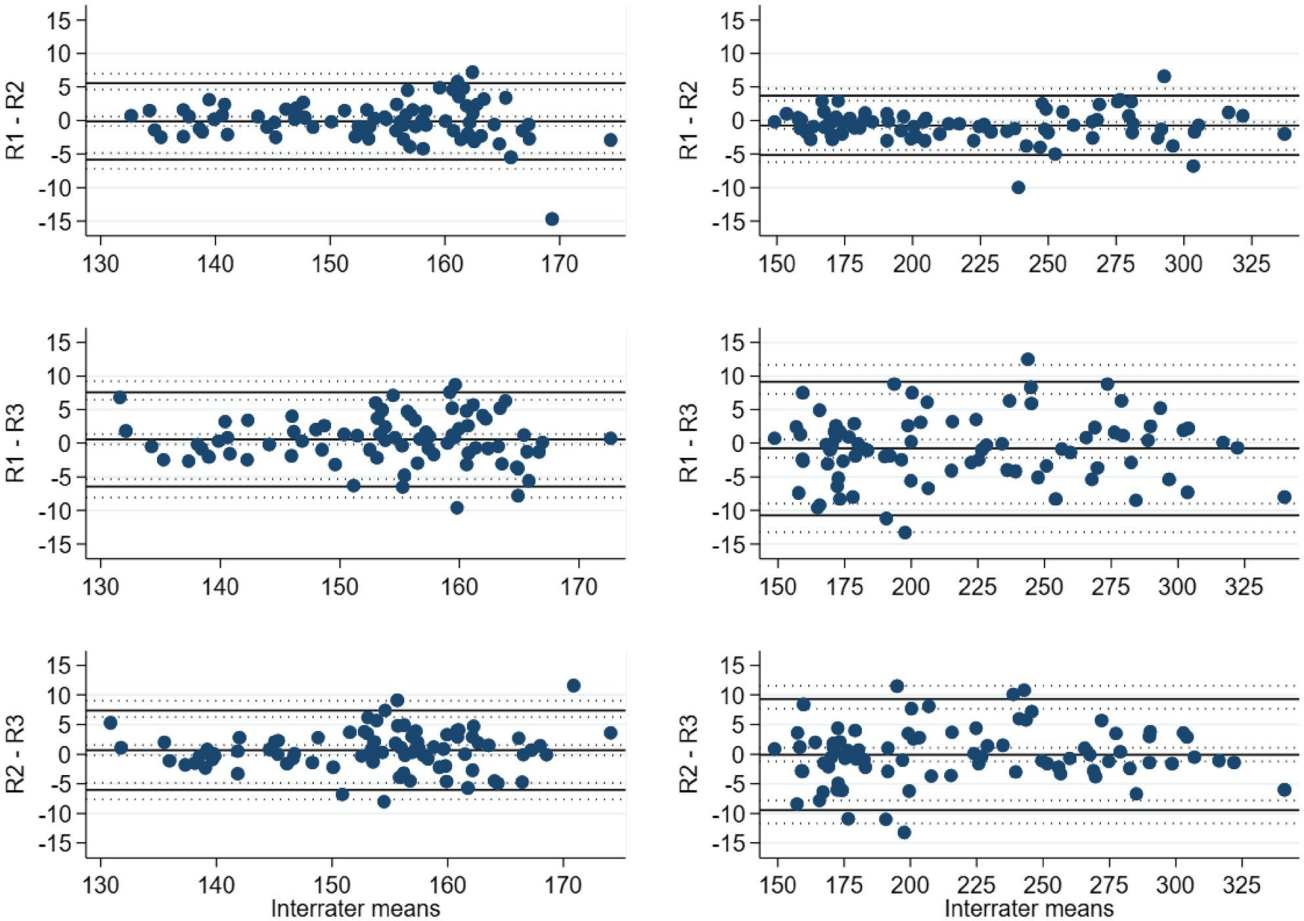
BA LoA plots showing the interrater agreement analysis for µCT-scans [mm^3^]. Left panel: control. Right panel: lymphedema. *BA LoA* Bland–Altman Limits of Agreement, *mm* millimeters, *R1* rater 1, *R2* rater 2, *R3* rater 3, *CT* computed tomography, *μ* micro.

The estimated mean difference for the lymphedema hindlimb between the rater 1 (R1), rater 2 (R2) and rater 3 (R3) was − 0.73 mm^3^ 95% CI [− 1.26, − 0.21], − 0.81 mm^3^ 95% CI [− 2.16, 0.55] and − 0.07 mm^3^ 95% CI [− 1.24, 1.09] for R1-R2, R1-R3 and R2-R3, respectively. For the control hindlimb the difference was − 0.13 mm^3^ 95% CI [− 0.85, 0.59], 0.56 mm^3^ 95% CI [− 0.22, 1.34] and 0.69 mm^3^ 95% CI [− 0.16, 1.53], respectively. The interrater agreement results for the µCT-scans are summarized in Table 1 and graphed in Fig. 4.

**Table 1:** Bias and BA LoA estimates with 95% CIs for interrater comparisons of µCT-scans.

Variable	Comparison	Group	Bias		BA LoA	
			Estimate	95% CI	Estimates	Outer 95% CI limits
µCT scans	R1–R2	Control	− 0.13	− 0.85, 0.59	− 5.84, 5.59	− 7.22, 6.97
		Lymphedema	− 0.73	− 1.26, − 0.21	− 5.17, 3.70	− 6.21, 4.74
	R1–R3	Control	0.56	− 0.22, 1.34	− 6.46, 7.58	− 8.07, 9.19
		Lymphedema	− 0.81	− 2.16, 0.55	− 10.74, 9.12	− 13.26, 11.64
	R2–R3	Control	0.69	− 0.16, 1.53	− 6.01, 7.39	− 7.63, 9.01
		Lymphedema	− 0.07	− 1.24, 1.09	− 9.44, 9.30	− 11.69, 11.55

Data is presented as estimated mean difference and respective 95% confidence interval in cubic millimeters (mm^3^), supplemented by Bland–Altman Limits of Agreement. Bland–Altman Limits of Agreement is the mean difference ± 1.96 standard deviation of the difference.

*BA* Bland–Altman, *LoA* Limits of Agreement, *CI* confidence interval, *R1* rater 1, *R2* rater 2, *R3* rater 3, *CT* computed tomography, *μ* micro.

#### Plethysmometer

**Figure 5:**
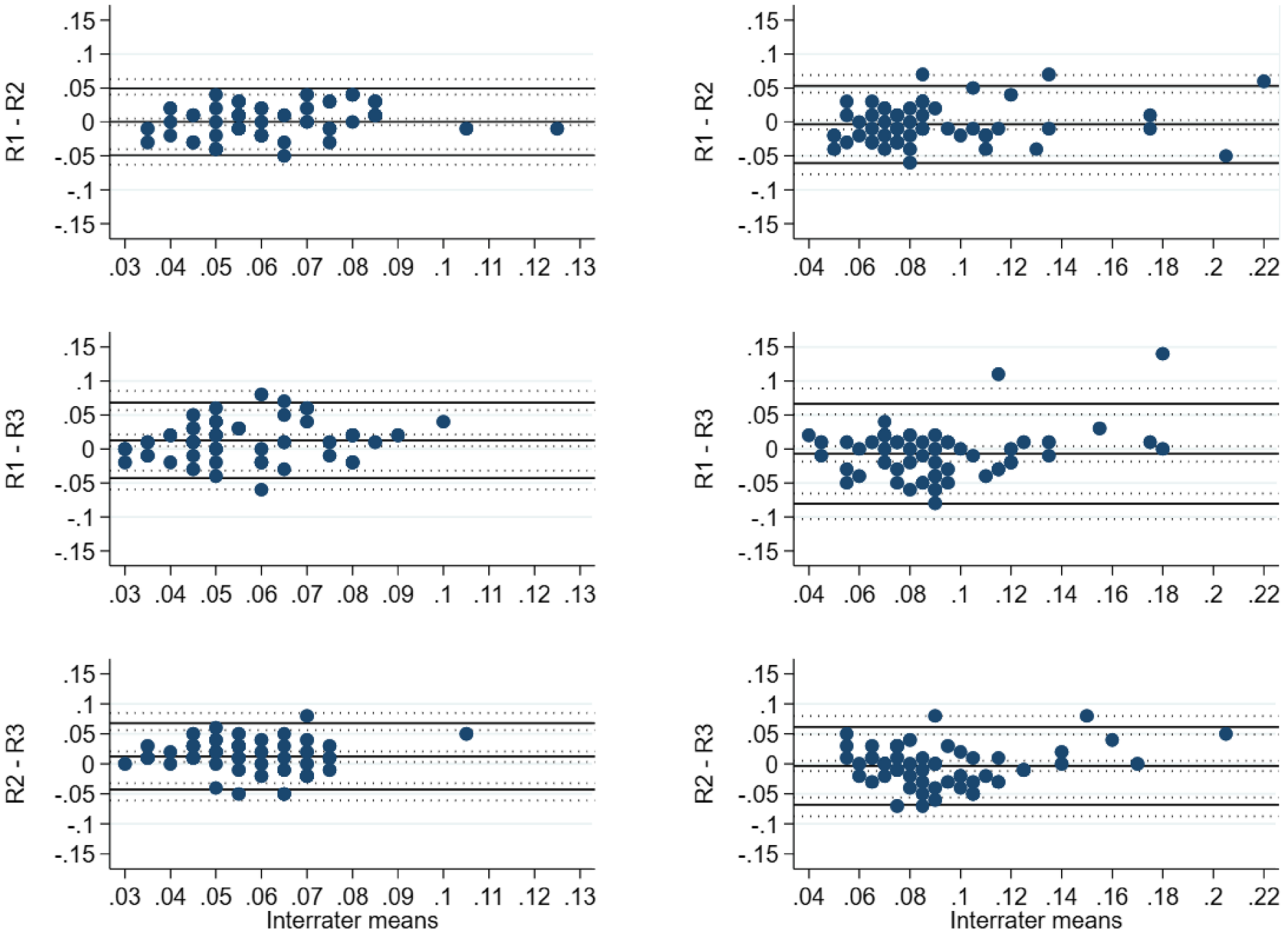
BA LoA plots showing the interrater agreement analysis for plethysmometer [ml]. Left panel: control. Right panel: lymphedema. *BA LoA* Bland–Altman Limits of Agreement, *ml* milliliters, *R1* rater 1, *R2* rater 2, *R3* rater 3.

The estimated mean difference for the lymphedema hindlimb between the raters (R1, R2, R3) was 0 ml 95% CI [− 0.01, 0.003], − 0.007 ml 95% CI [− 0.02, 0.004] and − 0.004 ml 95% CI [− 0.01, 0.005] for R1-R2, R1-R3 and R2-R3, respectively. For the control hindlimb the difference was 0 ml 95% CI − 0.005, 0.005], 0.01 ml 95% CI [0.004, 0.02] and 0.01 ml 95% CI [0.003, 0.02], respectively. The interrater agreement results for the plethysmometer are summarized in Table 2 and graphed in Fig. 5.

**Table 2:** Bias and BA LoA estimates with 95% CIs for interrater comparisons of the plethysmometer.

Variable	Comparison	Group	Bias		BA LoA	
			Estimate	95% CI	Estimates	Outer 95% CI limits
Plethysmometer	R1–R2	Control	0	− 0.005, 0.005	− 0.05, 0.05	− 0.06, 006
		Lymphedema	0	− 0.01, 0.003	− 0.06, 0.05	− 0.08, 0.07
	R1–R3	Control	0.01	0.004, 0.02	− 0.04, 0.07	− 0.06, 0.09
		Lymphedema	− 0.007	− 0.02, 0.004	− 0.08, 0.07	− 0.10, 0.09
	R2–R3	Control	0.01	0.003, 0.02	− 0.04, 0.07	− 0.06, 0.09
		Lymphedema	− 0.004	− 0.01, 0.005	− 0.07, 0.06	− 0.09, 0.08

Data is presented as estimated mean difference and respective 95% confidence interval in cubic milliliters (ml) supplemented by Bland–Altman Limits of Agreement. Bland–Altman Limits of Agreement is the mean difference ± 1.96 standard deviation of the difference.

*BA* Bland–Altman, *LoA* Limits of Agreement, *CI* confidence interval, *R1* rater 1, *R2* rater 2, *R3* rater 3.

#### Electronic caliper

**Figure 6:**
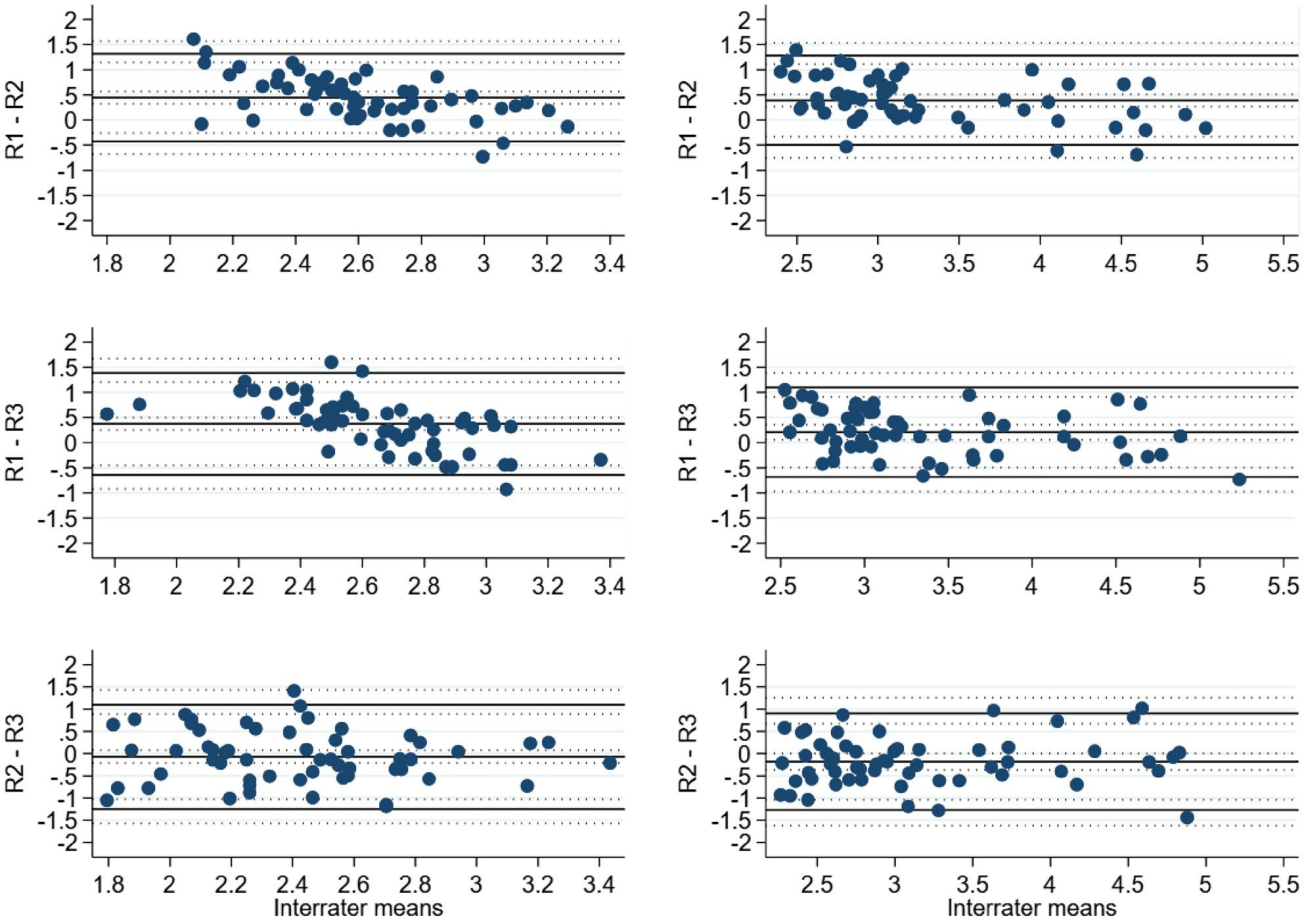
BA LoA plots showing the interrater agreement analysis for electronic caliper [mm]. Left panel: control. Right panel: lymphedema. *BA LoA* Bland–Altman Limits of Agreement, *mm* millimeters, *R1* rater 1, *R2* rater 2, *R3* rater 3.

The estimated mean difference for the lymphedema hindlimb between the raters (R1, R2, R3) was 0.39 mm 95% CI [0.27, 0.51], 0.21 mm 95% CI [0.06, 0.36] and − 0.18 mm 95% CI [− 0.37, 0] for R1-R2, R1-R3 and R2-R3, respectively. For the control hindlimb the difference was 0.45 mm 95% CI [0.33, 0.57], 0.37 mm 95% CI [0.25, 0.50] and − 0.07 mm 95% CI [− 0.21, 0.07], respectively. The interrater agreement results for the electronic caliper are summarized in Table 3 and graphed in Fig. 6.

**Table 3:** Bias and BA LoA estimates with 95% CIs for interrater comparisons of electronic caliper.

Variable	Comparison	Group	Bias		BA LoA	
			Estimate	95% CI	Estimates	Outer 95% CI limits
Electronic caliper	R1–R2	Control	0.45	0.33, 0.57	− 0.42, 1.32	− 0.68, 1.57
		Lymphedema	0.39	0.27, 0.51	− 0.50, 1.28	− 0.75, 1.54
	R1–R3	Control	0.37	0.25, 0.50	− 0.64, 1.39	− 0.92, 1.67
		Lymphedema	0.21	0.06, 0.36	− 0.68, 1.10	− 0.97, 1.39
	R2–R3	Control	− 0.07	− 0.21, 0.07	− 1.24, 1.10	− 1.57, 1.43
		Lymphedema	− 0.18	− 0.37, 0	− 1.27, 0.90	− 1.62, 1.26

Data is presented as estimated mean difference and respective 95% confidence interval in millimeters (mm) supplemented by Bland–Altman Limits of Agreement. Bland–Altman Limits of Agreement is the mean difference ± 1.96 standard deviation of the difference.

*BA* Bland–Altman, *LoA* Limits of Agreement, *CI* confidence interval, *R1* rater 1, *R2* rater 2, *R3* rater 3.

### Correlation analysis

#### µCT-scans vs. plethysmometer

The correlation coefficient between the µCT-scans and the plethysmometer for the lymphedema hindlimb was 0.56, 95% CI [0.42–0.70] (p < 0.0001) across all raters. The correlation coefficients were 0.65, 95% CI [0.44–0.86] (p < 0.0001), 0.70, 95% CI [0.54–0.87] (p < 0.0001) and 0.27, 95% CI [− 0.06 to 0.60] (p = 0.11) for raters R1, R2 and R3, respectively. For the control hindlimb the overall correlation coefficient was 0.11, 95% CI [− 0.04 to 0.25] (p = 0.15). The correlation coefficients were 0.23, 95% CI [0–0.46] (p = 0.05), − 0.05, 95% CI [− 0.31 to 0.20] (p = 0.69) and 0.10, 95% CI [− 0.18 to 0.38] (p = 0.50) for raters R1, R2 and R3, respectively.

#### µCT-scans vs. electronic caliper

The correlation coefficient between the µCT-scans and the electronic caliper for the lymphedema hindlimb was 0.85 95% CI [0.80–0.89] (p < 0.0001) across all raters. The correlation coefficients were 0.90, 95% CI [0.84–0.96] (p < 0.0001), 0.87, 95% CI [0.81–0.94] (p < 0.0001) and 0.84, 95% CI [0.76–0.92] (p < 0.0001), for raters R1, R2 and R3, respectively. For the control hindlimb the overall correlation coefficient was 0.04, 95% CI [− 0.12 to 0.19] (p = 0.64). The correlation coefficients were 0.22, 95% CI [0.02–0.42] (p = 0.033), 0, 95% CI [− 0.24 to 0.24] (p = 0.99) and − 0.01, 95% CI [− 0.31 to 0.29] (p = 0.96) for raters R1, R2 and R3, respectively.

The correlation coefficients are summarized in Table 4.

**Table 4:** Correlation coefficients with 95% CIs for plethysmometer and electronic caliper vs µCT-scans.

Comparison	Group	Rater	Correlation coefficient		
			Estimate	95% CI	P-value
µCT-scans vs. plethysmometer	Control hindlimb	All	0.11	− 0.04 to 0.25	0.15
		1	0.23	0–0.46	0.05
		2	− 0.05	− 0.31 to 0.20	0.69
		3	0.10	− 0.18 to 0.38	0.50
	Lymphedema hindlimb	All	0.56	0.42–0.70	< 0.0001
		1	0.65	0.44–0.86	< 0.0001
		2	0.70	0.54–0.87	< 0.0001
		3	0.27	− 0.06 to 0.60	0.11
µCT-scans vs. electronic caliper	Control hindlimb	All	0.04	− 0.12 to 0.19	0.64
		1	0.22	0.02–0.42	0.033
		2	0	− 0.24 to 0.24	0.99
		3	− 0.01	− 0.31 to 0.29	0.96
	Lymphedema hindlimb	All	0.85	0.80–0.89	< 0.0001
		1	0.90	0.84–0.96	< 0.0001
		2	0.87	0.81–0.94	< 0.0001
		3	0.84	0.76–0.92	< 0.0001

*CT* computed tomography, *μ* micro.

## Discussion

In this study, we examined the interrater agreement of plethysmometer, electronic caliper and µCT-scans in the measurement of lymphedema in mice. Subsequently, we did a correlation analysis between µCT-scans and the two conventional modalities (plethysmometer and electronic caliper). Twenty-four mice were included in this study. Lymphedema was induced by irradiation and surgery, and the mice were measured with µCT-scans, electronic caliper and plethysmometer in weeks 1, 2, 4 and 8 by three raters. The estimated mean difference for the hindlimbs between the three raters was then calculated.

The three different measurement modalities are discussed in three different paragraphs. The correlation analysis is likewise discussed in a separate paragraph.

### Plethysmometer

The mean interrater differences for the lymphedema hindlimb for the plethysmometer were 0 ml, − 0.007 ml and 0.004 ml between the three raters. The volumes of the hindlimbs ranged from 0.02 to 0.25 ml with the mean volume being 0.09 ml for the lymphedema hindlimb. Therefore, the highest mean interrater difference equals 7.78% of the mean hindlimb volume, and the lowest difference equals 0% of the mean volume.

The mean differences of 0 ml and 0.004 ml indicate that the plethysmometer has a low risk of measurement bias, while − 0.007 ml indicates a moderate risk of measurement bias.

Overall, the plethysmometer has a low to moderate risk of measurement bias.

The low range of volumes (75% of the lymphedema hindlimbs being 0.11 ml or less) should theoretically lead to low mean differences, which is the case in R1 vs R2 (0 ml) and R2 vs R3 (0.004 ml) but not the case in R1 vs R3 (− 0.007 ml). The low range of numbers was due to the small size of the mice and the plethysmometer’s lowest detectable difference (0.01 ml). The low range of numbers increases the risk of a biased low difference between the mean interrater differences, thus a biased high interrater agreement.

The lowest detectable difference was 0.01 ml in the plethysmometer that we used. 0.01 ml equals 10 mm^3^ and is a considerable amount in mice of this size, where the mean control hindlimb is 154 mm^3^ over 8 weeks. In contrast, the mean difference between rater R2 and R3 measured by the µCT was 0.07 mm^3^ equaling 0.00007 ml.

The size of the mice, and the water principle itself, made it difficult to standardize the measurements when inserting the hindlimb of the mice into the water. A few millimeters of deeper or shallower insertion into the water yielded 0.01 ml of difference. It is also important to note that every time a hindlimb is inserted and removed from the water, a small amount of water will adhere to the hindlimb of the mice, and therefore be removed from the plethysmometer. When water is removed the subsequent measurement will not take the new water level into account unless the plethysmometer is recalibrated. Ideally, the plethysmometer should be recalibrated after each measurement, a step the manufacturer only recommends at the beginning of the measurements. The full calibration took 20 min, which made calibration practically impossible after each measurement. Calibration was done every fifth mouse approximately, making measuring with the plethysmometer a lot more complicated than anticipated.

The plethysmometer is being used in lymphedema research in mice and was lastly used by Hayashida et al.^10^. They used a plethysmometer from Muromachi (MK-101 CMP; Muromachi Kikai Co., Ltd., Tokyo, Japan). According to the manufacturer, it can detect changes as low as 0.001 ml for mice and 0.01 ml for rats^18^. This is perhaps an overestimation due to the water principle. This assertion is backed by Shioiya et al.^11^. They studied lymphedema in the hindlimb of mice with a plethysmometer from Ugo Basile (Gemonio, Italy). This plethysmometer has, in agreement with the plethysmometer used in this study, a lowest detectable difference of 0.01 ml^19^. We found two other plethysmometers with 0.01 ml as the lowest detectable difference^20,21^, and one that did not classify^22^. We could not find a plethysmometer with the same claim regarding the 0.001 ml of accuracy. However, the plethysmometer from Muromachi should be examined as it can potentially be more sensitive than other plethysmometers from other manufacturers.

The plethysmometer is probably better suited for animals of a bigger size. Shejawal et al.^12^ assessed the plethysmometer in the hindlimb of 18 rats weighing 180–220 g. In comparison, our mice weighed approximately 20 g. They found a high correlation between the rat’s hindlimbs and different known volumes inserted in the water, but they did not examine the interrater agreement^12^. Further studies are needed to examine the interrater agreement of the plethysmometer in rats. The highest mean interrater differences for our control hindlimb, which did not have lymphedema and thus a lot smaller than the lymphedema hindlimb, was 17.2% compared to the lymphedema hindlimb of 7.8%. This indicates that the plethysmometer might have a smaller risk of measurement bias for bigger animals.

The water principle, which is used in the plethysmometer, was examined by Pan et al.^23^ on a mouse tail model. Pan et al. found highly variable measurements within the same tail resulting in a high risk of measurement bias^23^. The tail of mice should theoretically be better suited for a water displacement technique, as it is easily insertable in the small tube, can be easily standardized and has a lower risk of bias in terms of shallower/deeper insertion of the tail as the diameter is smaller than a hindlimb. Still, Pan et al. found highly variable measurements^23^. A plethysmometer is relatively expensive (less than a µCT-scanner), but once the plethysmometer is bought, there are no ongoing expenses.

### µCT-scans

The mean interrater differences for the µCT-scans for the lymphedema hindlimbs were − 0.73 mm^3^, − 0.81 mm^3^ and − 0.07 mm^3^ between the raters. The mean volume of the lymphedema hindlimb was 220.1 mm^3^. Therefore, the highest mean interrater difference equals 0.37% of the mean hindlimb volume, and the lowest difference equals 0.03% of the mean volume. This indicates an extremely low risk of measuring bias in agreement with our previous study^16^ and Frueh et al.^5^.

The mean differences for the control hindlimb were similar with the lowest being − 0.13 mm^3^ 95% CI [− 0.85, 0.59] between R1 and R3 and 0.69 mm^3^ 95% CI [− 0.16, 1.53] between R2 and R3 with 153.7 mm^3^ as the mean volume equaling 0.08% and 0.45%. The results of the control hindlimbs are in agreement with the lymphedema hindlimbs, and underlines the extremely low risk of measurement bias of the µCT-scans.

It should be noted that this study used Inveon Research workplace (version 4.2, IRW; Siemens Healthcare, Ballerup, Denmark) as the software for measuring the volume through the µCT-scans. It is unclear whether the same results can be obtained by different software. This should be investigated in future studies.

A µCT-scanner is the most expensive of the modalities, and there are ongoing expenses as each µCT-scan carry a cost.

### Electronic caliper

The mean interrater differences for the electronic caliper for the lymphedema hindlimbs were 0.39 mm, 0.21 mm and − 0.18 mm between the three raters. The mean thickness of the paw was 3.27 mm. Therefore, the highest mean interrater difference equals 11.92% of the mean paw thickness, and the lowest difference equals 5.50% of the mean paw thickness.

The results of the control hindlimbs are comparable with the lymphedema hindlimbs (2.27% lowest and 17.6% as the highest).

The mean differences between the raters are relatively low and indicate a low to moderate risk of measurement bias.

The use of an electronic caliper to measure the thickness of the paw is used as a surrogate parameter for hindlimb volume in lymphedema research on mice and was lastly used by Daneshgaran et al.^24^.

The electronic caliper is theoretically a good instrument in measuring the paw size in mice, as it can detect minimal changes in paw thickness of 0.01 mm.

It is the cheapest option of our three modalities as it costs less than the plethysmometer and a µCT-scanner, and there are no ongoing expenses once the caliper is bought. The caliper is easy to use.

A limitation is that the paw of the mice is soft and can be depressed by pressure of the caliper. Thus, it can be speculated that just a tiny amount of pressure can result in significant variations of results.

Frueh et al.^5^ assessed the electronic caliper in the mouse hindlimb and found a high interrater agreement, although not as high as µCT. Sharma et al.^25^ compared the electronic caliper vs a plethysmometer from Ugo Basile (Gemonio, Italy) on the hindlimb of mice and found the caliper to be more sensitive. No interrater agreement was examined. The electronic caliper is also being used in the mice lymphedema tail models, as seen in Ghanta et al.^26^, but the interrater agreement is yet to be examined.

### Correlation analysis

µCT had previously shown an extremely low risk of measurements bias^5,16^ and was for that reason, chosen as the reference standard in the correlation analysis. The overall correlation coefficient between the plethysmometer and the µCT-scans was 0.56, 95% CI [0.42–0.70] (p < 0.0001), indicating a moderate correlation.

The result of the electronic caliper vs µCT-scans was 0.85 95% CI [0.80–0.89] (p < 0.0001) indicating a strong correlation. These results indicate that the electronic caliper is superior to the plethysmometer.

The control hindlimb showed no correlation for raters combined across both modalities. The control hindlimb is significantly smaller than the lymphedema hindlimb. This highlights the issue of measuring small volumes with the plethysmometer and electronic caliper.

It is important to note that in order to do the correlation analysis, we had to standardize the measurements of the three different modalities, and therefore there is a risk of bias. Because of this risk, our conclusion is solely based upon the interrater agreement.

## Conclusion

**Figure 7:**
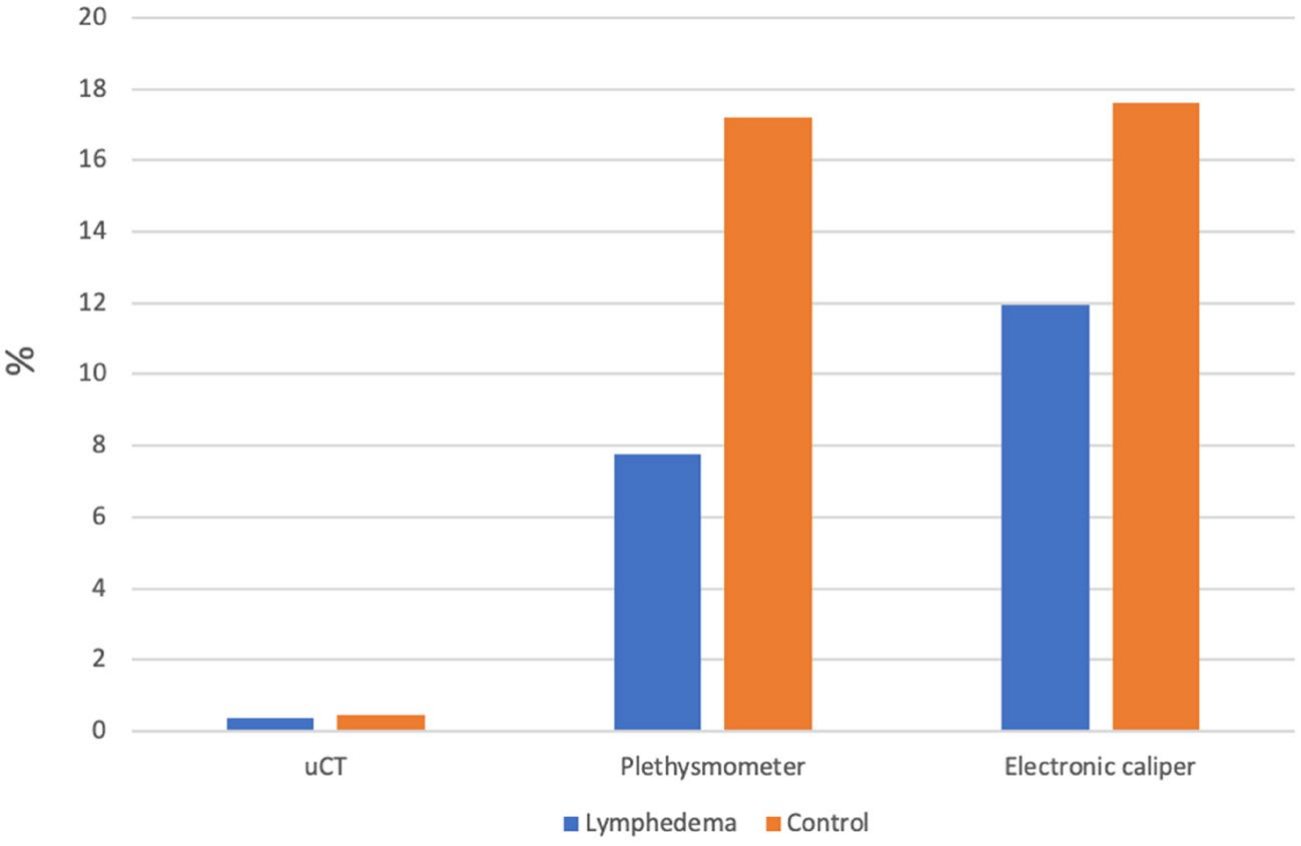
Graph comparing the percentages of the highest interrater differences divided by the mean hindlimb measurements across all three modalities. *L* lymphedema hindlimb, *C* control hindlimb, *CT* computed tomography, *μ* micro.

µCT-scans were superior to the electronic caliper and the plethysmometer when assessing lymphedema in the hindlimb of mice. Electronic caliper was comparable to the plethysmometer, both having a low to moderate risk of measurement bias. See Table 5 and Fig. 7.

**Table 5:** Table c﻿omparing the lowest and highest interrater differences, mean hindlimb measurements and the percentage between the two across all three modalities.

Modality	Lowest mean interrater difference	Highest mean interrater difference	Mean hindlimb volume	Lowest %	Highest %
μCT (L)	− 0.07 (R2 − R3)	− 0.81 (R1 − R3)	220.1 mm^3^	0.03	0.37
μCT (C)	− 0.13 (R1 − R2)	0.69 (R2 − R3)	153.7 mm^3^	0.08	0.45
Plethysmometer (L)	0 (R1 − R2)	0.007 (R1 − R3)	0.09 ml	0.00	7.78
Plethysmometer (C)	0 (R1 − R2)	0.01 (R1 − R3)	0.058 ml	0.00	17.2
Electronic caliper (L)	− 0.18 (R2 − R3)	0.39 (R1 − R2)	3.27 mm	5.50	11.93
Electronic caliper (C)	− 0.07 (R2 − R3)	0.45 (R1 − R2)	2.55 mm	2.27	17.6

*L* lymphedema hindlimb, *C* control hindlimb, *CT* computed tomography, *μ* micro.

The plethysmometer had a low range of values due to the lowest detectable difference of 0.01 ml and the small size of the hindlimb of the mice. This increased the risk of a biased high interrater agreement. Therefore, the plethysmometer should not be used in rodents of this size to assess the lymphedema of the hindlimbs. The plethysmometer might be a better instrument in rodents of a bigger size. This should be investigated in future studies.

The electronic caliper should only be used if there is no µCT-scanner available. The µCT-scans have an extremely low risk of measurement bias in the assessment of hindlimb lymphedema in mice and should be used whenever possible.

## Materials and methods

The mice were measured with µCT-scans, electronic caliper and plethysmometer in weeks 1, 2, 4 and 8 by three raters R1 (AB), R2 (AW) and R3 (FD).

All three raters measured all mice at all weeks. The absolute number of samples was, therefore, 24 mice * 4 weeks = 96 samples. The CLSI recommendation is at least 40 samples^27^. Week numbers 1 and 8 were chosen to get the broadest range of values. The absolute number of samples and the broad range of values are in accordance with the guidelines from CLSI^27^. Measurements were conducted independently and were blinded between raters. The ARRIVE guidelines^28^ were followed to the extent of possibility, as there are natural limitations to the guidelines as this is not an experimental study.

Plethysmometer and electronic caliper measurements in week 1 are unavailable as the machine administering the anesthetic gases did not work properly, and measurements while the mice were awake, were assessed not sufficient. Measurements in weeks 2, 4 and 8 were conducted properly. We had prior experience with µCT and these were done with anesthesia injections all weeks. Although the results from week 1 were not available we still had 57 samples in week 2, 4 and 8 which is in accordance with the The Clinical and Laboratory Standard Institute’s (CLSI) recommendations, recommending at least 40 samples^27^.

### Animals

The National Animal Inspectorate in Denmark approved this study (2018-15-0201-01445), which includes an ethical approval. All experiments were conducted according to the national laws of animal research.

Twenty-four 9-week old female C57BL6 mice from Janvier (Janvier Labs, Le Genest-Saint-Isle, Saint-Berthevin Cedex, France) were used in this study. The mice were acclimatized for 7 days preoperatively.

Postoperatively the mice were housed individually and received oral analgesic treatment (Buprenorphine, 0.2 mg/g) daily for 3 days. They were maintained at a normal 12-h day/night cycle at 21 degrees Celsius with a humidity of 45–55%. They were fed a standard diet and water ad libitum. The mice were euthanized by cervical dislocation under anesthesia at the end of the study.

During the study, four mice were euthanized for ethical reasons by the veterinarian due to poor wound healing. One died during anesthesia while being µCT-scanned.

### Lymphedema model

The mice had lymphedema induced with a previously described model^29^ with minor modifications.

Briefly, the lymphedema was established in four separate procedures. Irradiation two times before surgery, surgery itself and irradiation after surgery.

The hindlimbs of the mice were irradiated with a dose of 22.5 Gray (Gy) in three fractions (Gulmay D3100 X-ray instrument (Xstrahl, Camberley, UK) with a dose rate of 5.11 Gy/min (100 kVp, 10 mA, HVL 2.53 Al). The irradiation was performed 7 and 3 days before the surgery and 3 days after surgery.

The surgery consisted of microsurgery of the right hindlimb. The lymph vessels were tied with 10-0 suture and two lymph nodes were dissected. The surgical and irradiation details are explained and shown in a video by Wiinholt et al.^29^.

The modifications consisted of irradiation with three fractions of 7.5 Gy instead of two fractions of 10 Gy.

### µCT-scans

**Figure 8:**
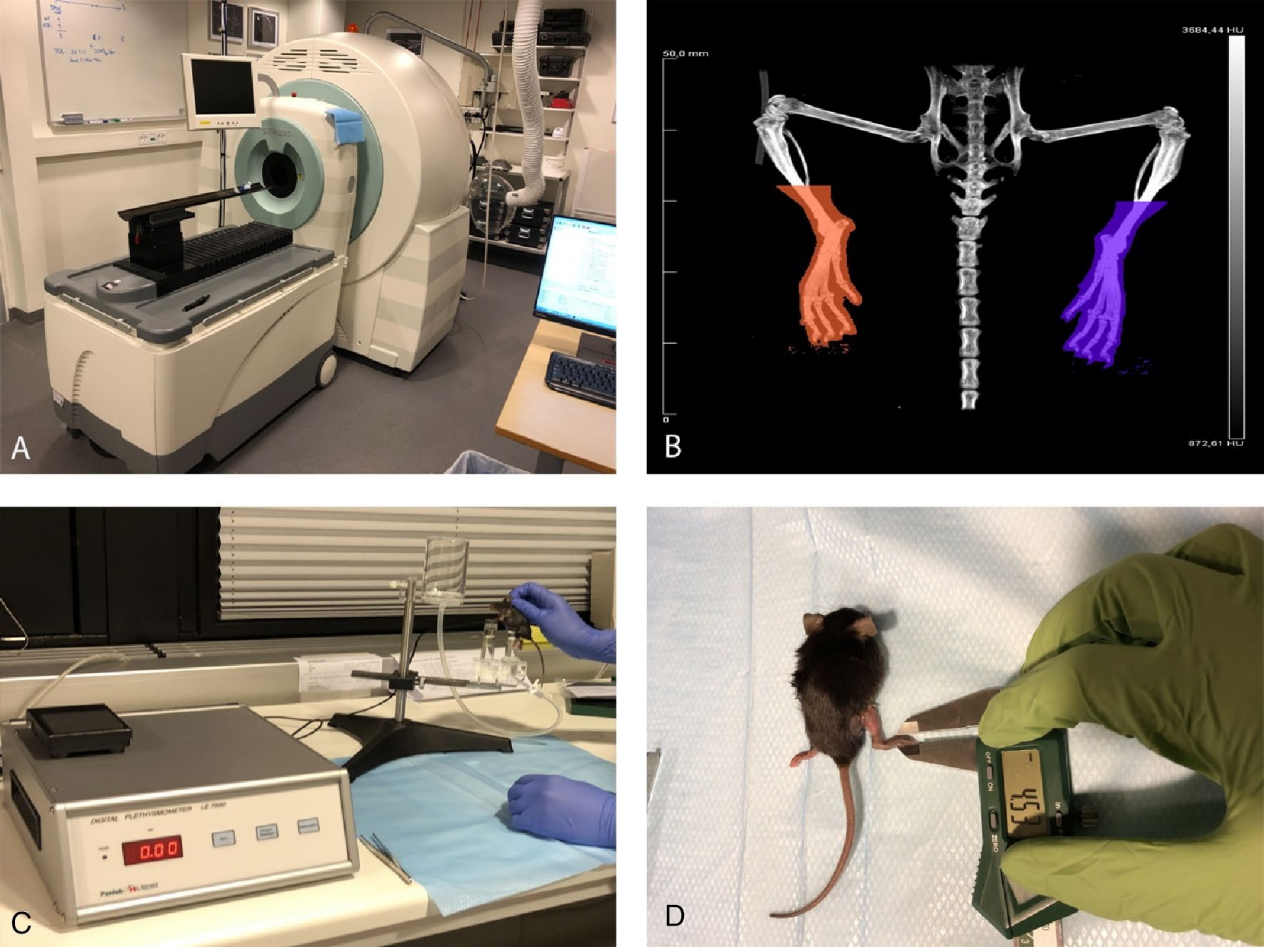
Overview of the modalities. (**A**) μ-CT scanner, (**B**) μ-CT scan showing the volumetric measures of both hindlimbs, (**C**) Plethysmometer measurement showing the inserting of the right lymphedema hindlimb, (**D**) Electronic caliper measurement of right lymphedema hindlimb paw. *CT* computed tomography, *μ* micro.

The µCT-scans were performed on a Siemens INVEON multimodality pre-clinical scanner (Siemens pre-clinical solutions, Knoxville, TN, USA) (Fig. 8). The animals were anesthetized with 1.5–2% isoflurane (ScanVet Animal Health, Fredensborg, Denmark) mixed with 100% oxygen during the scans. The mice were placed front feet first in prone position on a heated animal bed (38 mm).

The standardization, assessment and analysis of the µCT-scans have previously been described^16^ Briefly; the distal tibiofibular joint was chosen as the upper volumetric boundary limit. The volume was then calculated in Inveon research workplace software, version 4.2 (IRW; Siemens Healthcare, Ballerup, Denmark). See Fig. 8.

### Plethysmometer

The plethysmometer was model LE7500 from Panlab (Panlab S.L.U/Harvard Apparatus, Barcelona, Spain). The animals were anesthetized with 1.5–2% isoflurane (ScanVet Animal Health, Fredensborg, Denmark) mixed with 100% oxygen during the measurements.

To standardize the measurements, the musculotendinous junction of the gastrocnemius muscle was used as a landmark in the plethysmometer measurements^10^. The hindlimb of the mice was inserted in the water until the junction. The volumetric measurements were then noted.

### Electronic caliper

The electronic caliper used was from Insize Digital Caliper Series 1108 (Insize Co. LTD, China). The animals were anesthetized with 1.5–2% isoflurane (ScanVet Animal Health, Fredensborg, Denmark) mixed with 100% oxygen during the measurements. The paw thickness was measured in a transverse technique between the first and second proximal pad of the paw with the electronic caliper. See Fig. 8.

### Raters

None of the raters had prior experience with the plethysmometer or the electronic caliper. All raters had experience with the µCT-scans. R1 (AB) had approximately 25 h of experience while R2 (AW) and R3 (FD) had approximately 50 h of experience. The plethysmometer and electronic caliper techniques require no prior training, while the µCT-scan require a basic understanding of CT imaging e.g., a Bachelor of Science in Medicine and approximately 1 h of training. All raters were blinded between each other’s measurements.

### Statistics

Descriptive statistics were done according to data type, i.e. median and range were shown for continuous variables, frequencies and respective percentages for categorical variables. Visual presentations of continuous variables comprised boxplots. Interrater agreement was assessed with Bland–Altman Limits of Agreement (BA LoA)^30,31^, adjusted for longitudinal correlated data as the mice were measured up to four times^32,33^. The estimated bias (i.e. the mean difference), as well as the BA LoA were supplemented by respective 95% confidence intervals (95% CI). Regarding the latter, only the outer 95% CI limits are of clinical interest for interpretation purposes^34^. Comparisons between the two conventional measuring techniques and the µ-CT were done by Bravais-Pearson correlation coefficients on the original scales, supplemented by bootstrapped 95% CIs. Standardization of all modalities’ measurements was explored in order to enable comparison of measurements from different scales; however, neither this endeavor did imply satisfactory agreement (see Supplemental Figs. 1, 2). All analyses were done with STATA/MP 17.0 (StataCorp, College Station, Texas 77845 USA).

## Supplementary Information

Supplementary Information.
