# Supplementary material for: Microcomputed tomography versus plethysmometer and electronic caliper in the measurements of lymphedema in the hindlimb of mice
Source: Sci Rep. 2022 Jul 18;12:12267. doi: 10.1038/s41598-022-16311-2 (PMC9293915; doi:10.1038/s41598-022-16311-2)
Supplement: Supplementary file 1 — Supplementary Information. [file 41598_2022_16311_MOESM1_ESM.docx]

Supplemental figure 1


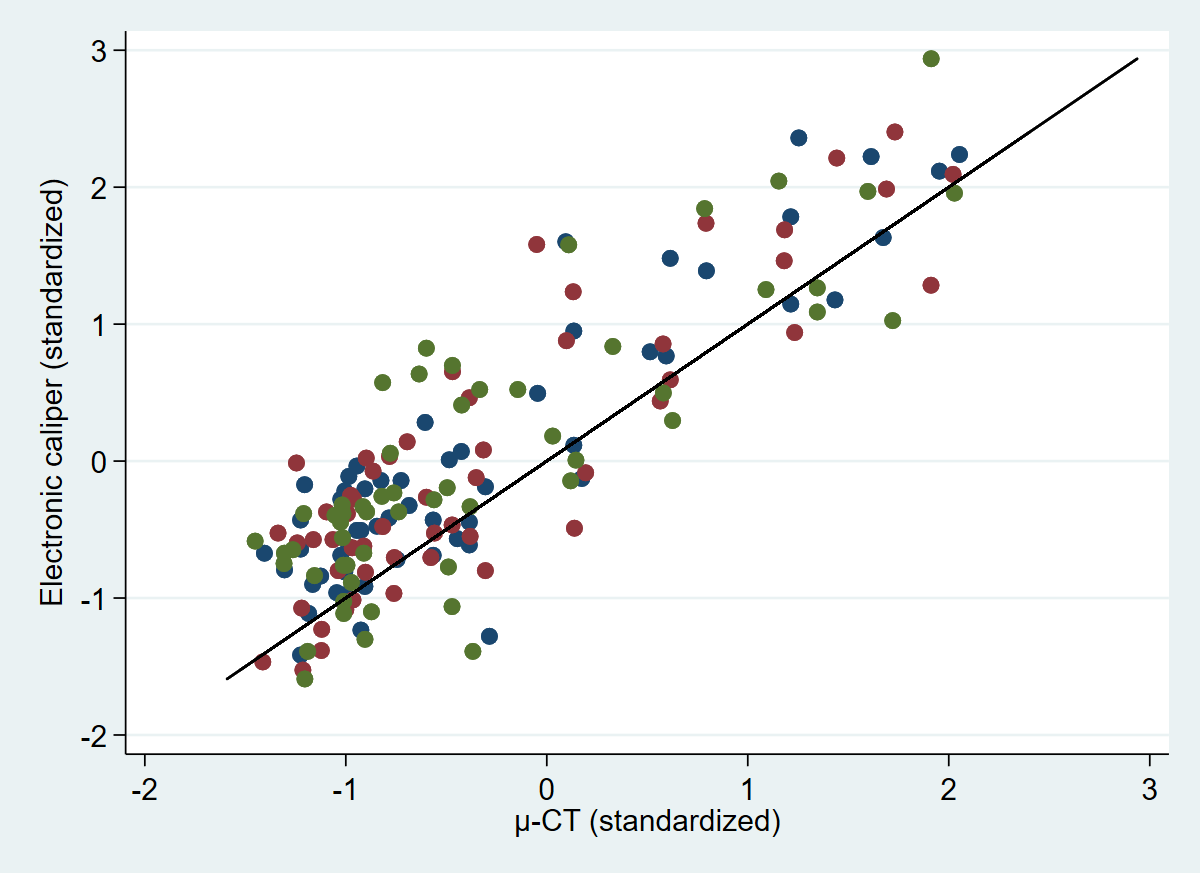


Supplemental figure 2


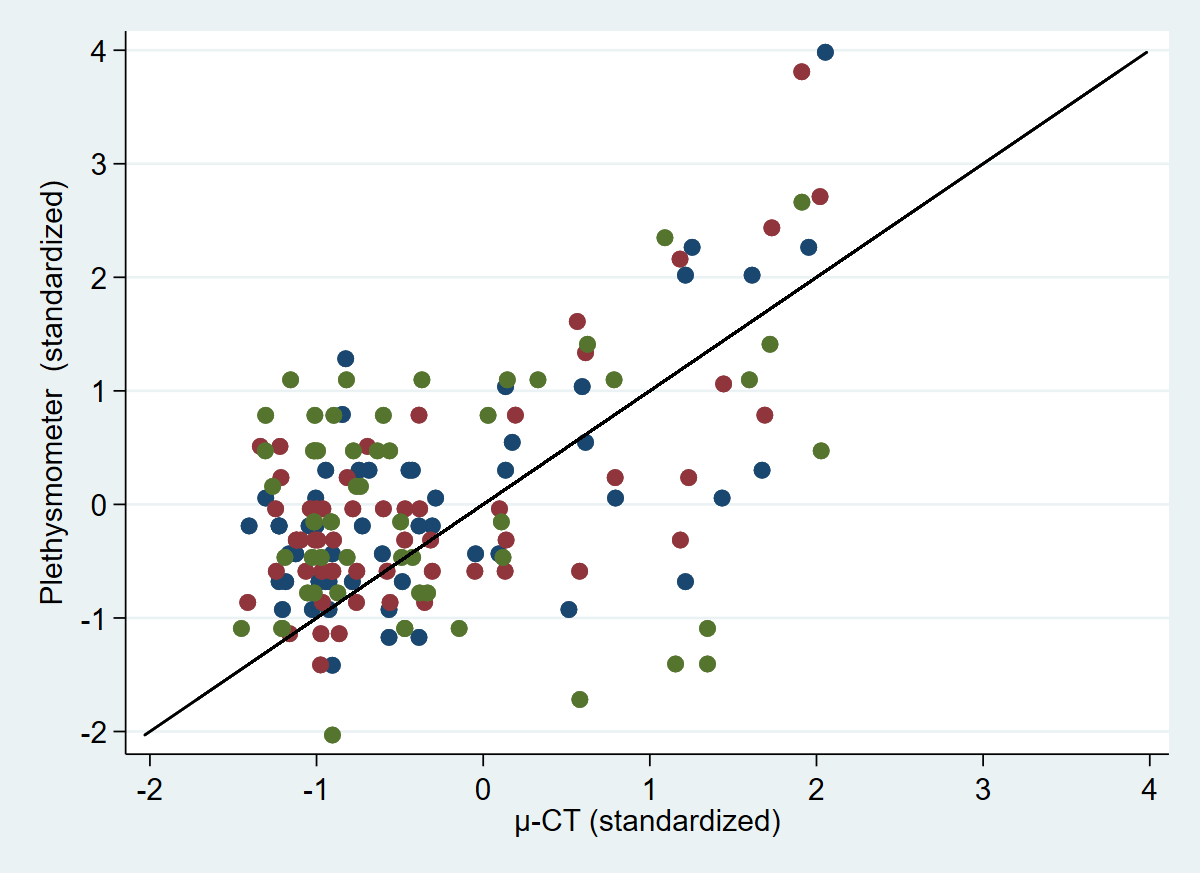


Supplemental table 1

| Week 1 |  | Lymphedema | Control | Week 2 |  | Lymphedema | Control |
| --- | --- | --- | --- | --- | --- | --- | --- |
|  | Mouse 1 | 278 | 164 |  | Mouse 1 | 280 | 164 |
|  | Mouse 2 | 305 | 161 |  | Mouse 2 |  |  |
|  | Mouse 3 | 294 | 162 |  | Mouse 3 | 245 | 152 |
|  | Mouse 4 | 209 | 148 |  | Mouse 4 | 180 | 135 |
|  | Mouse 5 | 240 | 158 |  | Mouse 5 | 195 | 158 |
|  | Mouse 6 | 296 | 163 |  | Mouse 6 | 291 | 162 |
|  | Mouse 7 | 213 | 160 |  | Mouse 7 | 180 | 158 |
|  | Mouse 8 | 256 | 134 |  | Mouse 8 | 259 | 133 |
|  | Mouse 9 | 234 | 145 |  | Mouse 9 | 217 | 155 |
|  | Mouse 10 | 234 | 141 |  | Mouse 10 | 224 | 150 |
|  | Mouse 11 | 336 | 157 |  | Mouse 11 |  |  |
|  | Mouse 12 | 248 | 136 |  | Mouse 12 | 174 | 138 |
|  | Mouse 13 | 277 | 138 |  | Mouse 13 | 303 | 148 |
|  | Mouse 14 | 249 | 148 |  | Mouse 14 | 205 | 155 |
|  | Mouse 15 | 281 | 163 |  | Mouse 15 | 200 | 162 |
|  | Mouse 16 | 203 | 142 |  | Mouse 16 |  |  |
|  | Mouse 17 | 250 | 149 |  | Mouse 17 | 191 | 147 |
|  | Mouse 18 | 289 | 147 |  | Mouse 18 | 300 | 154 |
|  | Mouse 19 | 237 | 134 |  | Mouse 19 | 200 | 138 |
|  | Mouse 20 | 265 | 164 |  | Mouse 20 | 282 | 166 |
|  | Mouse 21 | 266 | 152 |  | Mouse 21 | 249 | 163 |
|  | Mouse 22 | 270 | 160 |  | Mouse 22 | 228 | 164 |
|  | Mouse 23 | 268 | 152 |  | Mouse 23 | 317 | 158 |
|  | Mouse 24 | 221 | 144 |  | Mouse 24 |  |  |
| Week 4 |  | Lymphedema | Control | Week 8 |  | Lymphedema | Control |
|  | Mouse 1 | 191 | 148 |  | Mouse 1 | 168 | 152 |
|  | Mouse 2 |  |  |  | Mouse 2 |  |  |
|  | Mouse 3 | 169 | 151 |  | Mouse 3 | 169 | 163 |
|  | Mouse 4 |  |  |  | Mouse 4 |  |  |
|  | Mouse 5 | 170 | 155 |  | Mouse 5 | 168 | 161 |
|  | Mouse 6 | 280 | 166 |  | Mouse 6 | 189 | 173 |
|  | Mouse 7 | 177 | 156 |  | Mouse 7 | 161 | 161 |
|  | Mouse 8 | 226 | 138 |  | Mouse 8 | 158 | 141 |
|  | Mouse 9 | 178 | 163 |  | Mouse 9 | 163 | 160 |
|  | Mouse 10 | 174 | 154 |  | Mouse 10 | 158 | 156 |
|  | Mouse 11 |  |  |  | Mouse 11 |  |  |
|  | Mouse 12 | 154 | 144 |  | Mouse 12 | 149 | 140 |
|  | Mouse 13 | 250 | 144 |  | Mouse 13 | 173 | 156 |
|  | Mouse 14 | 160 | 155 |  | Mouse 14 | 159 | 155 |
|  | Mouse 15 | 182 | 159 |  | Mouse 15 | 169 | 158 |
|  | Mouse 16 |  |  |  | Mouse 16 |  |  |
|  | Mouse 17 | 183 | 157 |  | Mouse 17 | 167 | 159 |
|  | Mouse 18 | 185 | 153 |  | Mouse 18 | 173 | 163 |
|  | Mouse 19 | 158 | 140 |  | Mouse 19 |  |  |
|  | Mouse 20 | 197 | 155 |  | Mouse 20 | 172 | 158 |
|  | Mouse 21 | 198 | 166 |  | Mouse 21 | 170 | 157 |
|  | Mouse 22 | 204 | 167 |  | Mouse 22 | 172 | 162 |
|  | Mouse 23 | 322 | 165 |  | Mouse 23 | 226 | 167 |
|  | Mouse 24 |  |  |  | Mouse 24 |  |  |

Supplemental table 2

| Week 1 |  | Lymphedema | Control | Week 2 |  | Lymphedema | Control |
| --- | --- | --- | --- | --- | --- | --- | --- |
|  | Mouse 1 |  |  |  | Mouse 1 | 0.06 | 0.03 |
|  | Mouse 2 |  |  |  | Mouse 2 |  |  |
|  | Mouse 3 |  |  |  | Mouse 3 | 0.05 | 0.03 |
|  | Mouse 4 |  |  |  | Mouse 4 |  |  |
|  | Mouse 5 |  |  |  | Mouse 5 | 0.06 | 0.03 |
|  | Mouse 6 |  |  |  | Mouse 6 | 0.09 | 0.04 |
|  | Mouse 7 |  |  |  | Mouse 7 | 0.06 | 0.04 |
|  | Mouse 8 |  |  |  | Mouse 8 | 0.09 | 0.05 |
|  | Mouse 9 |  |  |  | Mouse 9 | 0.07 | 0.04 |
|  | Mouse 10 |  |  |  | Mouse 10 | 0.07 | 0.05 |
|  | Mouse 11 |  |  |  | Mouse 11 |  |  |
|  | Mouse 12 |  |  |  | Mouse 12 | 0.03 | 0.03 |
|  | Mouse 13 |  |  |  | Mouse 13 | 0.1 | 0.03 |
|  | Mouse 14 |  |  |  | Mouse 14 | 0.09 | 0.06 |
|  | Mouse 15 |  |  |  | Mouse 15 | 0.04 | 0.02 |
|  | Mouse 16 |  |  |  | Mouse 16 |  |  |
|  | Mouse 17 |  |  |  | Mouse 17 | 0.04 | 0.03 |
|  | Mouse 18 |  |  |  | Mouse 18 | 0.17 | 0.03 |
|  | Mouse 19 |  |  |  | Mouse 19 | 0.08 | 0.06 |
|  | Mouse 20 |  |  |  | Mouse 20 | 0.18 | 0.09 |
|  | Mouse 21 |  |  |  | Mouse 21 | 0.13 | 0.09 |
|  | Mouse 22 |  |  |  | Mouse 22 | 0.11 | 0.09 |
|  | Mouse 23 |  |  |  | Mouse 23 | 0.18 | 0.12 |
|  | Mouse 24 |  |  |  | Mouse 24 |  |  |
| Week 4 |  | Lymphedema | Control | Week 8 |  | Lymphedema | Control |
|  | Mouse 1 | 0.05 | 0.07 |  | Mouse 1 | 0.08 | 0.04 |
|  | Mouse 2 |  |  |  | Mouse 2 |  |  |
|  | Mouse 3 | 0.08 | 0.07 |  | Mouse 3 | 0.09 | 0.09 |
|  | Mouse 4 |  |  |  | Mouse 4 |  |  |
|  | Mouse 5 | 0.06 | 0.07 |  | Mouse 5 | 0.05 | 0.05 |
|  | Mouse 6 | 0.17 | 0.07 |  | Mouse 6 | 0.07 | 0.07 |
|  | Mouse 7 | 0.12 | 0.07 |  | Mouse 7 | 0.07 | 0.05 |
|  | Mouse 8 | 0.1 | 0.05 |  | Mouse 8 | 0.08 | 0.08 |
|  | Mouse 9 | 0.14 | 0.07 |  | Mouse 9 | 0.07 | 0.05 |
|  | Mouse 10 | 0.07 | 0.07 |  | Mouse 10 | 0.06 | 0.03 |
|  | Mouse 11 |  |  |  | Mouse 11 |  |  |
|  | Mouse 12 | 0.09 | 0.07 |  | Mouse 12 | 0.08 | 0.06 |
|  | Mouse 13 | 0.11 | 0.05 |  | Mouse 13 | 0.06 | 0.07 |
|  | Mouse 14 | 0.06 | 0.06 |  | Mouse 14 | 0.05 | 0.05 |
|  | Mouse 15 | 0.1 | 0.08 |  | Mouse 15 | 0.08 | 0.1 |
|  | Mouse 16 |  |  |  | Mouse 16 |  |  |
|  | Mouse 17 | 0.08 | 0.06 |  | Mouse 17 | 0.08 | 0.1 |
|  | Mouse 18 | 0.1 | 0.09 |  | Mouse 18 | 0.05 | 0.07 |
|  | Mouse 19 | 0.08 | 0.1 |  | Mouse 19 | 0.04 | 0.05 |
|  | Mouse 20 | 0.1 | 0.09 |  | Mouse 20 | 0.1 | 0.05 |
|  | Mouse 21 | 0.1 | 0.1 |  | Mouse 21 | 0.06 | 0.05 |
|  | Mouse 22 | 0.08 | 0.1 |  | Mouse 22 | 0.06 | 0.06 |
|  | Mouse 23 | 0.25 | 0.1 |  | Mouse 23 | 0.13 | 0.05 |
|  | Mouse 24 |  |  |  | Mouse 24 |  |  |

Supplemental table 3

| Week 1 |  | Lymphedema | Control | Week 2 |  | Lymphedema | Control |
| --- | --- | --- | --- | --- | --- | --- | --- |
|  | Mouse 1 |  |  |  | Mouse 1 | 4.65 | 2.96 |
|  | Mouse 2 |  |  |  | Mouse 2 |  |  |
|  | Mouse 3 |  |  |  | Mouse 3 | 4 | 3.31 |
|  | Mouse 4 |  |  |  | Mouse 4 |  |  |
|  | Mouse 5 |  |  |  | Mouse 5 | 3.48 | 3.24 |
|  | Mouse 6 |  |  |  | Mouse 6 | 4.25 | 2.83 |
|  | Mouse 7 |  |  |  | Mouse 7 | 3.2 | 2.86 |
|  | Mouse 8 |  |  |  | Mouse 8 | 4.39 | 2.6 |
|  | Mouse 9 |  |  |  | Mouse 9 | 3.8 | 3.2 |
|  | Mouse 10 |  |  |  | Mouse 10 | 4.53 | 2.84 |
|  | Mouse 11 |  |  |  | Mouse 11 |  |  |
|  | Mouse 12 |  |  |  | Mouse 12 | 3.34 | 2.9 |
|  | Mouse 13 |  |  |  | Mouse 13 | 4.55 | 2.64 |
|  | Mouse 14 |  |  |  | Mouse 14 | 2.63 | 2.82 |
|  | Mouse 15 |  |  |  | Mouse 15 | 3.18 | 2.72 |
|  | Mouse 16 |  |  |  | Mouse 16 |  |  |
|  | Mouse 17 |  |  |  | Mouse 17 | 3.02 | 2.64 |
|  | Mouse 18 |  |  |  | Mouse 18 | 4.94 | 2.65 |
|  | Mouse 19 |  |  |  | Mouse 19 | 3.07 | 2.81 |
|  | Mouse 20 |  |  |  | Mouse 20 | 5.03 | 3.2 |
|  | Mouse 21 |  |  |  | Mouse 21 | 3.98 | 2.86 |
|  | Mouse 22 |  |  |  | Mouse 22 | 3.39 | 2.74 |
|  | Mouse 23 |  |  |  | Mouse 23 | 4.87 | 2.79 |
|  | Mouse 24 |  |  |  | Mouse 24 |  |  |
| Week 4 |  | Lymphedema | Control | Week 8 |  | Lymphedema | Control |
|  | Mouse 1 | 3.19 | 2.54 |  | Mouse 1 | 3.02 | 3.12 |
|  | Mouse 2 |  |  |  | Mouse 2 |  |  |
|  | Mouse 3 | 2.83 | 2.69 |  | Mouse 3 | 3.33 | 2.93 |
|  | Mouse 4 |  |  |  | Mouse 4 |  |  |
|  | Mouse 5 | 3.4 | 2.06 |  | Mouse 5 | 3.29 | 3.03 |
|  | Mouse 6 | 4.23 | 2.63 |  | Mouse 6 | 3.66 | 2.91 |
|  | Mouse 7 | 3.16 | 2.97 |  | Mouse 7 | 2.88 | 2.79 |
|  | Mouse 8 | 4.1 | 2.77 |  | Mouse 8 | 2.54 | 2.59 |
|  | Mouse 9 | 3.38 | 3.28 |  | Mouse 9 | 2.92 | 2.71 |
|  | Mouse 10 | 2.87 | 3 |  | Mouse 10 | 3.19 | 2.75 |
|  | Mouse 11 |  |  |  | Mouse 11 |  |  |
|  | Mouse 12 | 2.95 | 3.05 |  | Mouse 12 | 3.03 | 2.68 |
|  | Mouse 13 | 4.45 | 3.17 |  | Mouse 13 | 2.66 | 2.64 |
|  | Mouse 14 | 2.74 | 2.72 |  | Mouse 14 | 3.36 | 2.63 |
|  | Mouse 15 | 3 | 2.83 |  | Mouse 15 | 2.94 | 2.61 |
|  | Mouse 16 |  |  |  | Mouse 16 |  |  |
|  | Mouse 17 | 3.38 | 2.83 |  | Mouse 17 | 2.84 | 2.4 |
|  | Mouse 18 | 3.26 | 2.81 |  | Mouse 18 | 3.14 | 2.78 |
|  | Mouse 19 | 3.05 | 2.81 |  | Mouse 19 | 2.79 | 2.26 |
|  | Mouse 20 | 3.1 | 2.73 |  | Mouse 20 | 3.45 | 2.88 |
|  | Mouse 21 | 3.52 | 3.1 |  | Mouse 21 | 3.06 | 2.85 |
|  | Mouse 22 | 3.35 | 2.94 |  | Mouse 22 | 3.14 | 2.75 |
|  | Mouse 23 | 4.95 | 3.3 |  | Mouse 23 | 3.55 | 2.96 |
|  | Mouse 24 |  |  |  | Mouse 24 |  |  |

Supplemental table 4

| Week 1 |  | Lymphedema | Control | Week 2 |  | Lymphedema | Control |
| --- | --- | --- | --- | --- | --- | --- | --- |
|  | Mouse 1 | 274.9 | 158.2 |  | Mouse 1 | 279.3 | 159.2 |
|  | Mouse 2 | 305.7 | 164.1 |  | Mouse 2 |  |  |
|  | Mouse 3 | 297.8 | 162.3 |  | Mouse 3 | 249 | 154.7 |
|  | Mouse 4 | 211 | 147.5 |  | Mouse 4 | 181.1 | 133.5 |
|  | Mouse 5 | 243.8 | 158.1 |  | Mouse 5 | 196.5 | 156.4 |
|  | Mouse 6 | 289.4 | 159.4 |  | Mouse 6 | 292.3 | 164.3 |
|  | Mouse 7 | 213.5 | 162.8 |  | Mouse 7 | 180.8 | 158 |
|  | Mouse 8 | 254.7 | 136.5 |  | Mouse 8 | 259.7 | 132.3 |
|  | Mouse 9 | 244 | 145.3 |  | Mouse 9 | 217.5 | 154.5 |
|  | Mouse 10 | 235.6 | 140.2 |  | Mouse 10 | 224.9 | 150.2 |
|  | Mouse 11 | 338 | 157.9 |  | Mouse 11 |  |  |
|  | Mouse 12 | 249.3 | 138.4 |  | Mouse 12 | 174.4 | 139.2 |
|  | Mouse 13 | 274.2 | 136.4 |  | Mouse 13 | 304.7 | 146.1 |
|  | Mouse 14 | 246.5 | 149 |  | Mouse 14 | 204.7 | 154.7 |
|  | Mouse 15 | 281.5 | 168.5 |  | Mouse 15 | 202.4 | 157.1 |
|  | Mouse 16 | 206 | 139.6 |  | Mouse 16 |  |  |
|  | Mouse 17 | 255 | 146.3 |  | Mouse 17 | 191.1 | 146.8 |
|  | Mouse 18 | 291.6 | 145.3 |  | Mouse 18 | 306.8 | 152.4 |
|  | Mouse 19 | 238.2 | 135.4 |  | Mouse 19 | 200.9 | 137.4 |
|  | Mouse 20 | 267.6 | 161.6 |  | Mouse 20 | 279.2 | 158.8 |
|  | Mouse 21 | 266.2 | 152.9 |  | Mouse 21 | 250.8 | 161.9 |
|  | Mouse 22 | 267.6 | 161.5 |  | Mouse 22 | 229.7 | 164.6 |
|  | Mouse 23 | 267.9 | 153.9 |  | Mouse 23 | 315.8 | 158.3 |
|  | Mouse 24 | 224 | 143.4 |  | Mouse 24 |  |  |
| Week 4 |  | Lymphedema | Control | Week 8 |  | Lymphedema | Control |
|  | Mouse 1 | 190 | 147.6 |  | Mouse 1 | 166.7 | 150.5 |
|  | Mouse 2 |  |  |  | Mouse 2 |  |  |
|  | Mouse 3 | 171.2 | 153.4 |  | Mouse 3 | 169.4 | 160.8 |
|  | Mouse 4 |  |  |  | Mouse 4 |  |  |
|  | Mouse 5 | 170.3 | 156.5 |  | Mouse 5 | 165.1 | 163.6 |
|  | Mouse 6 | 281.8 | 167.5 |  | Mouse 6 | 192 | 175.9 |
|  | Mouse 7 | 176.7 | 160.2 |  | Mouse 7 | 163.8 | 163 |
|  | Mouse 8 | 226.6 | 139.7 |  | Mouse 8 | 157.6 | 137.9 |
|  | Mouse 9 | 179.1 | 158.4 |  | Mouse 9 | 163.9 | 160.1 |
|  | Mouse 10 | 171.1 | 153.7 |  | Mouse 10 | 159.2 | 156.2 |
|  | Mouse 11 |  |  |  | Mouse 11 |  |  |
|  | Mouse 12 | 153 | 145 |  | Mouse 12 | 149.2 | 142.1 |
|  | Mouse 13 | 248.3 | 146.5 |  | Mouse 13 | 174.8 | 156.7 |
|  | Mouse 14 | 161.8 | 154.8 |  | Mouse 14 | 158.9 | 158.9 |
|  | Mouse 15 | 182 | 157.6 |  | Mouse 15 | 171.8 | 158.4 |
|  | Mouse 16 |  |  |  | Mouse 16 |  |  |
|  | Mouse 17 | 181.9 | 154.6 |  | Mouse 17 | 168 | 154.5 |
|  | Mouse 18 | 185.2 | 153.9 |  | Mouse 18 | 175 | 166.5 |
|  | Mouse 19 | 157.8 | 139.8 |  | Mouse 19 |  |  |
|  | Mouse 20 | 196.4 | 157.8 |  | Mouse 20 | 171.5 | 158.7 |
|  | Mouse 21 | 200.7 | 168.7 |  | Mouse 21 | 169.9 | 156.2 |
|  | Mouse 22 | 204.2 | 167.6 |  | Mouse 22 | 171.7 | 176.7 |
|  | Mouse 23 | 321.3 | 161.8 |  | Mouse 23 | 226.9 | 163.6 |
|  | Mouse 24 |  |  |  | Mouse 24 |  |  |

Supplemental table 5

| Week 1 |  | Lymphedema | Control | Week 2 |  | Lymphedema | Control |
| --- | --- | --- | --- | --- | --- | --- | --- |
|  | Mouse 1 |  |  |  | Mouse 1 | 0.08 | 0.06 |
|  | Mouse 2 |  |  |  | Mouse 2 |  |  |
|  | Mouse 3 |  |  |  | Mouse 3 | 0.07 | 0.07 |
|  | Mouse 4 |  |  |  | Mouse 4 |  |  |
|  | Mouse 5 |  |  |  | Mouse 5 | 0.09 | 0.07 |
|  | Mouse 6 |  |  |  | Mouse 6 | 0.13 | 0.09 |
|  | Mouse 7 |  |  |  | Mouse 7 | 0.09 | 0.09 |
|  | Mouse 8 |  |  |  | Mouse 8 | 0.1 | 0.07 |
|  | Mouse 9 |  |  |  | Mouse 9 | 0.07 | 0.06 |
|  | Mouse 10 |  |  |  | Mouse 10 | 0.09 | 0.04 |
|  | Mouse 11 |  |  |  | Mouse 11 |  |  |
|  | Mouse 12 |  |  |  | Mouse 12 | 0.07 | 0.05 |
|  | Mouse 13 |  |  |  | Mouse 13 | 0.12 | 0.07 |
|  | Mouse 14 |  |  |  | Mouse 14 | 0.07 | 0.06 |
|  | Mouse 15 |  |  |  | Mouse 15 | 0.06 | 0.05 |
|  | Mouse 16 |  |  |  | Mouse 16 |  |  |
|  | Mouse 17 |  |  |  | Mouse 17 | 0.07 | 0.06 |
|  | Mouse 18 |  |  |  | Mouse 18 | 0.18 | 0.07 |
|  | Mouse 19 |  |  |  | Mouse 19 | 0.09 | 0.09 |
|  | Mouse 20 |  |  |  | Mouse 20 | 0.17 | 0.08 |
|  | Mouse 21 |  |  |  | Mouse 21 | 0.14 | 0.06 |
|  | Mouse 22 |  |  |  | Mouse 22 | 0.12 | 0.08 |
|  | Mouse 23 |  |  |  | Mouse 23 | 0.23 | 0.13 |
|  | Mouse 24 |  |  |  | Mouse 24 |  |  |
| Week 4 |  | Lymphedema | Control | Week 8 |  | Lymphedema | Control |
|  | Mouse 1 | 0.09 | 0.07 |  | Mouse 1 | 0.07 | 0.04 |
|  | Mouse 2 |  |  |  | Mouse 2 |  |  |
|  | Mouse 3 | 0.05 | 0.05 |  | Mouse 3 | 0.08 | 0.05 |
|  | Mouse 4 |  |  |  | Mouse 4 |  |  |
|  | Mouse 5 | 0.08 | 0.06 |  | Mouse 5 | 0.08 | 0.05 |
|  | Mouse 6 | 0.1 | 0.07 |  | Mouse 6 | 0.06 | 0.04 |
|  | Mouse 7 | 0.05 | 0.06 |  | Mouse 7 | 0.08 | 0.06 |
|  | Mouse 8 | 0.07 | 0.03 |  | Mouse 8 | 0.09 | 0.08 |
|  | Mouse 9 | 0.1 | 0.08 |  | Mouse 9 | 0.08 | 0.08 |
|  | Mouse 10 | 0.04 | 0.04 |  | Mouse 10 | 0.1 | 0.04 |
|  | Mouse 11 |  |  |  | Mouse 11 |  |  |
|  | Mouse 12 | 0.11 | 0.08 |  | Mouse 12 | 0.06 | 0.05 |
|  | Mouse 13 | 0.15 | 0.07 |  | Mouse 13 | 0.07 | 0.03 |
|  | Mouse 14 | 0.05 | 0.04 |  | Mouse 14 | 0.11 | 0.06 |
|  | Mouse 15 | 0.07 | 0.06 |  | Mouse 15 | 0.09 | 0.06 |
|  | Mouse 16 |  |  |  | Mouse 16 |  |  |
|  | Mouse 17 | 0.06 | 0.05 |  | Mouse 17 | 0.09 | 0.06 |
|  | Mouse 18 | 0.11 | 0.08 |  | Mouse 18 | 0.08 | 0.05 |
|  | Mouse 19 | 0.07 | 0.07 |  | Mouse 19 | 0.06 | 0.06 |
|  | Mouse 20 | 0.08 | 0.06 |  | Mouse 20 | 0.07 | 0.04 |
|  | Mouse 21 | 0.12 | 0.07 |  | Mouse 21 | 0.09 | 0.07 |
|  | Mouse 22 | 0.08 | 0.06 |  | Mouse 22 | 0.06 | 0.05 |
|  | Mouse 23 | 0.19 | 0.11 |  | Mouse 23 | 0.08 | 0.03 |
|  | Mouse 24 |  |  |  | Mouse 24 |  |  |

Supplemental table 6

| Week 1 |  | Lymphedema | Control | Week 2 |  | Lymphedema | Control |
| --- | --- | --- | --- | --- | --- | --- | --- |
|  | Mouse 1 |  |  |  | Mouse 1 | 4.5 | 2.99 |
|  | Mouse 2 |  |  |  | Mouse 2 |  |  |
|  | Mouse 3 |  |  |  | Mouse 3 | 3.8 | 2.96 |
|  | Mouse 4 |  |  |  | Mouse 4 |  |  |
|  | Mouse 5 |  |  |  | Mouse 5 | 3.63 | 2.96 |
|  | Mouse 6 |  |  |  | Mouse 6 | 4.94 | 3.29 |
|  | Mouse 7 |  |  |  | Mouse 7 | 3.11 | 2.11 |
|  | Mouse 8 |  |  |  | Mouse 8 | 4.54 | 2.8 |
|  | Mouse 9 |  |  |  | Mouse 9 | 4.41 | 3.33 |
|  | Mouse 10 |  |  |  | Mouse 10 | 3.82 | 2.13 |
|  | Mouse 11 |  |  |  | Mouse 11 |  |  |
|  | Mouse 12 |  |  |  | Mouse 12 | 2.56 | 2.19 |
|  | Mouse 13 |  |  |  | Mouse 13 | 4.75 | 2.84 |
|  | Mouse 14 |  |  |  | Mouse 14 | 2.41 | 2.29 |
|  | Mouse 15 |  |  |  | Mouse 15 | 2.98 | 2.2 |
|  | Mouse 16 |  |  |  | Mouse 16 |  |  |
|  | Mouse 17 |  |  |  | Mouse 17 | 2.49 | 2.42 |
|  | Mouse 18 |  |  |  | Mouse 18 | 5.1 | 2.56 |
|  | Mouse 19 |  |  |  | Mouse 19 | 2.62 | 2.22 |
|  | Mouse 20 |  |  |  | Mouse 20 | 4.31 | 2.72 |
|  | Mouse 21 |  |  |  | Mouse 21 | 3.58 | 2.63 |
|  | Mouse 22 |  |  |  | Mouse 22 | 3.01 | 2.56 |
|  | Mouse 23 |  |  |  | Mouse 23 | 4.16 | 1.9 |
|  | Mouse 24 |  |  |  | Mouse 24 |  |  |
| Week 4 |  | Lymphedema | Control | Week 8 |  | Lymphedema | Control |
|  | Mouse 1 | 2.86 | 2.33 |  | Mouse 1 | 2.6 | 2.13 |
|  | Mouse 2 |  |  |  | Mouse 2 |  |  |
|  | Mouse 3 | 2.87 | 2.06 |  | Mouse 3 | 2.77 | 2.07 |
|  | Mouse 4 |  |  |  | Mouse 4 |  |  |
|  | Mouse 5 | 2.76 | 2.14 |  | Mouse 5 | 2.77 | 2.46 |
|  | Mouse 6 | 3.87 | 3.36 |  | Mouse 6 | 2.64 | 1.91 |
|  | Mouse 7 | 3.02 | 2.69 |  | Mouse 7 | 1.92 | 1.44 |
|  | Mouse 8 | 4.12 | 2.41 |  | Mouse 8 | 3.07 | 2.56 |
|  | Mouse 9 | 2.68 | 2.42 |  | Mouse 9 | 2.05 | 1.97 |
|  | Mouse 10 | 2.87 | 2.18 |  | Mouse 10 | 1.8 | 1.69 |
|  | Mouse 11 |  |  |  | Mouse 11 |  |  |
|  | Mouse 12 | 2.64 | 2.49 |  | Mouse 12 | 1.85 | 1.54 |
|  | Mouse 13 | 3.45 | 2.94 |  | Mouse 13 | 2.4 | 1.74 |
|  | Mouse 14 | 2.6 | 2.45 |  | Mouse 14 | 2.18 | 1.96 |
|  | Mouse 15 | 2.49 | 2.26 |  | Mouse 15 | 2.85 | 2.58 |
|  | Mouse 16 |  |  |  | Mouse 16 |  |  |
|  | Mouse 17 | 2.27 | 2.49 |  | Mouse 17 | 2.41 | 2.07 |
|  | Mouse 18 | 3.2 | 2.6 |  | Mouse 18 | 3.1 | 2.42 |
|  | Mouse 19 | 2.58 | 2.36 |  | Mouse 19 | 2.47 | 2.27 |
|  | Mouse 20 | 2.69 | 2.85 |  | Mouse 20 | 2.55 | 1.27 |
|  | Mouse 21 | 3.47 | 2.69 |  | Mouse 21 | 2.17 | 2.05 |
|  | Mouse 22 | 3.15 | 2.6 |  | Mouse 22 | 2.23 | 2.18 |
|  | Mouse 23 | 4.84 | 3.11 |  | Mouse 23 | 2.67 | 1.82 |
|  | Mouse 24 |  |  |  | Mouse 24 |  |  |

Supplemental table 7

| Week 1 |  | Lymphedema | Control | Week 2 |  | Lymphedema | Control |
| --- | --- | --- | --- | --- | --- | --- | --- |
|  | Mouse 1 | 269.2 | 160.4 |  | Mouse 1 | 278.9 | 155.3 |
|  | Mouse 2 | 302.8 | 168.8 |  | Mouse 2 | 298.1 | 159.4 |
|  | Mouse 3 | 299.4 | 159.4 |  | Mouse 3 | 250.1 | 150.9 |
|  | Mouse 4 | 202.9 | 154.3 |  | Mouse 4 | 177.1 | 128.2 |
|  | Mouse 5 | 233.7 | 153.3 |  | Mouse 5 | 197.5 | 154.6 |
|  | Mouse 6 | 290.8 | 158.2 |  | Mouse 6 | 288.5 | 162.8 |
|  | Mouse 7 | 217.1 | 158.8 |  | Mouse 7 | 180.1 | 157 |
|  | Mouse 8 | 256.9 | 134.5 |  | Mouse 8 | 260.4 | 131.2 |
|  | Mouse 9 | 238 | 146.9 |  | Mouse 9 | 213.8 | 154.2 |
|  | Mouse 10 | 234.1 | 143.5 |  | Mouse 10 | 226.5 | 147.4 |
|  | Mouse 11 | 344 | 158.7 |  | Mouse 11 |  |  |
|  | Mouse 12 | 242.1 | 138.7 |  | Mouse 12 | 172.3 | 140.1 |
|  | Mouse 13 | 275.4 | 138.2 |  | Mouse 13 | 301.1 | 144 |
|  | Mouse 14 | 240.7 | 151.2 |  | Mouse 14 | 201.9 | 152.6 |
|  | Mouse 15 | 283.9 | 168.6 |  | Mouse 15 | 199.8 | 156.8 |
|  | Mouse 16 | 209.7 | 138.8 |  | Mouse 16 |  |  |
|  | Mouse 17 | 258.3 | 147 |  | Mouse 17 | 204.3 | 146.7 |
|  | Mouse 18 | 288.6 | 145.3 |  | Mouse 18 | 307.3 | 152.7 |
|  | Mouse 19 | 241.2 | 136.5 |  | Mouse 19 | 197.4 | 138.9 |
|  | Mouse 20 | 270.4 | 158.3 |  | Mouse 20 | 275.7 | 160.8 |
|  | Mouse 21 | 265.2 | 154.2 |  | Mouse 21 | 252.4 | 166.8 |
|  | Mouse 22 | 267.7 | 161.5 |  | Mouse 22 | 228.3 | 159.9 |
|  | Mouse 23 | 271.7 | 153 |  | Mouse 23 | 316.9 | 150.9 |
|  | Mouse 24 | 223.9 | 140.6 |  | Mouse 24 |  |  |
| Week 4 |  | Lymphedema | Control | Week 8 |  | Lymphedema | Control |
|  | Mouse 1 | 192.9 | 149 |  | Mouse 1 | 168.2 | 158.5 |
|  | Mouse 2 |  |  |  | Mouse 2 |  |  |
|  | Mouse 3 | 177.3 | 149.7 |  | Mouse 3 | 175.4 | 163.5 |
|  | Mouse 4 |  |  |  | Mouse 4 |  |  |
|  | Mouse 5 | 175.2 | 155.4 |  | Mouse 5 | 163.1 | 161.7 |
|  | Mouse 6 | 288.5 | 164.8 |  | Mouse 6 | 191 | 172.3 |
|  | Mouse 7 | 176.1 | 151.1 |  | Mouse 7 | 170.2 | 158.9 |
|  | Mouse 8 | 227 | 140 |  | Mouse 8 | 160.4 | 140.2 |
|  | Mouse 9 | 179.9 | 155.4 |  | Mouse 9 | 155.5 | 159.2 |
|  | Mouse 10 | 182 | 153.6 |  | Mouse 10 | 155.6 | 150 |
|  | Mouse 11 |  |  |  | Mouse 11 |  |  |
|  | Mouse 12 | 161.4 | 144.2 |  | Mouse 12 | 148.3 | 141.6 |
|  | Mouse 13 | 237.5 | 144.2 |  | Mouse 13 | 170.4 | 151 |
|  | Mouse 14 | 169.6 | 151.3 |  | Mouse 14 | 157.7 | 164.6 |
|  | Mouse 15 | 182.9 | 162.2 |  | Mouse 15 | 170 | 156.4 |
|  | Mouse 16 |  |  |  | Mouse 16 |  |  |
|  | Mouse 17 | 184.1 | 157.8 |  | Mouse 17 | 170.1 | 159 |
|  | Mouse 18 | 196.2 | 157.8 |  | Mouse 18 | 175.7 | 166.6 |
|  | Mouse 19 | 160.7 | 139.7 |  | Mouse 19 |  |  |
|  | Mouse 20 | 202.6 | 158 |  | Mouse 20 | 170.3 | 153.8 |
|  | Mouse 21 | 189.2 | 167.3 |  | Mouse 21 | 170.4 | 156.4 |
|  | Mouse 22 | 196.5 | 166.9 |  | Mouse 22 | 171.2 | 165.1 |
|  | Mouse 23 | 322.7 | 166.3 |  | Mouse 23 | 222.5 | 160.7 |
|  | Mouse 24 |  |  |  | Mouse 24 |  |  |

Supplemental table 8

| Week 1 |  | Lymphedema | Control | Week 2 |  | Lymphedema | Control |
| --- | --- | --- | --- | --- | --- | --- | --- |
|  | Mouse 1 |  |  |  | Mouse 1 | 0.05 | 0.03 |
|  | Mouse 2 |  |  |  | Mouse 2 |  |  |
|  | Mouse 3 |  |  |  | Mouse 3 | 0.04 | 0.03 |
|  | Mouse 4 |  |  |  | Mouse 4 |  |  |
|  | Mouse 5 |  |  |  | Mouse 5 | 0.06 | 0.06 |
|  | Mouse 6 |  |  |  | Mouse 6 | 0.05 | 0.05 |
|  | Mouse 7 |  |  |  | Mouse 7 | 0.08 | 0.06 |
|  | Mouse 8 |  |  |  | Mouse 8 | 0.13 | 0.07 |
|  | Mouse 9 |  |  |  | Mouse 9 | 0.06 | 0.03 |
|  | Mouse 10 |  |  |  | Mouse 10 | 0.09 | 0.03 |
|  | Mouse 11 |  |  |  | Mouse 11 |  |  |
|  | Mouse 12 |  |  |  | Mouse 12 | 0.08 | 0.05 |
|  | Mouse 13 |  |  |  | Mouse 13 | 0.13 | 0.04 |
|  | Mouse 14 |  |  |  | Mouse 14 | 0.07 | 0.04 |
|  | Mouse 15 |  |  |  | Mouse 15 | 0.08 | 0.04 |
|  | Mouse 16 |  |  |  | Mouse 16 |  |  |
|  | Mouse 17 |  |  |  | Mouse 17 | 0.07 | 0.07 |
|  | Mouse 18 |  |  |  | Mouse 18 | 0.14 | 0.04 |
|  | Mouse 19 |  |  |  | Mouse 19 | 0.06 | 0.04 |
|  | Mouse 20 |  |  |  | Mouse 20 | 0.17 | 0.07 |
|  | Mouse 21 |  |  |  | Mouse 21 | 0.14 | 0.04 |
|  | Mouse 22 |  |  |  | Mouse 22 | 0.13 | 0.07 |
|  | Mouse 23 |  |  |  | Mouse 23 | 0.18 | 0.08 |
|  | Mouse 24 |  |  |  | Mouse 24 |  |  |
| Week 4 |  | Lymphedema | Control | Week 8 |  | Lymphedema | Control |
|  | Mouse 1 | 0.11 | 0.04 |  | Mouse 1 | 0.07 | 0.03 |
|  | Mouse 2 |  |  |  | Mouse 2 |  |  |
|  | Mouse 3 | 0.07 | 0.04 |  | Mouse 3 | 0.09 | 0.07 |
|  | Mouse 4 |  |  |  | Mouse 4 |  |  |
|  | Mouse 5 | 0.09 | 0.06 |  | Mouse 5 | 0.13 | 0.04 |
|  | Mouse 6 | 0.06 | 0.03 |  | Mouse 6 | 0.12 | 0.09 |
|  | Mouse 7 | 0.12 | 0.03 |  | Mouse 7 | 0.07 | 0.05 |
|  | Mouse 8 | 0.08 | 0.03 |  | Mouse 8 | 0.06 | 0.02 |
|  | Mouse 9 | 0.13 | 0.03 |  | Mouse 9 | 0.11 | 0.04 |
|  | Mouse 10 | 0.11 | 0.09 |  | Mouse 10 | 0.12 | 0.09 |
|  | Mouse 11 |  |  |  | Mouse 11 |  |  |
|  | Mouse 12 | 0.08 | 0.06 |  | Mouse 12 | 0.06 | 0.03 |
|  | Mouse 13 | 0.13 | 0.08 |  | Mouse 13 | 0.11 | 0.08 |
|  | Mouse 14 | 0.08 | 0.03 |  | Mouse 14 | 0.1 | 0.05 |
|  | Mouse 15 | 0.1 | 0.07 |  | Mouse 15 | 0.11 | 0.08 |
|  | Mouse 16 |  |  |  | Mouse 16 |  |  |
|  | Mouse 17 | 0.1 | 0.06 |  | Mouse 17 | 0.09 | 0.08 |
|  | Mouse 18 | 0.09 | 0.05 |  | Mouse 18 | 0.03 | 0.02 |
|  | Mouse 19 | 0.06 | 0.02 |  | Mouse 19 | 0.05 | 0.04 |
|  | Mouse 20 | 0.13 | 0.08 |  | Mouse 20 | 0.09 | 0.04 |
|  | Mouse 21 | 0.11 | 0.04 |  | Mouse 21 | 0.12 | 0.05 |
|  | Mouse 22 | 0.08 | 0.04 |  | Mouse 22 | 0.11 | 0.04 |
|  | Mouse 23 | 0.11 | 0.03 |  | Mouse 23 | 0.12 | 0.07 |
|  | Mouse 24 |  |  |  | Mouse 24 |  |  |

Supplemental table 9

| Week 1 |  | Lymphedema | Control | Week 2 |  | Lymphedema | Control |
| --- | --- | --- | --- | --- | --- | --- | --- |
|  | Mouse 1 |  |  |  | Mouse 1 | 4.89 | 2.58 |
|  | Mouse 2 |  |  |  | Mouse 2 |  |  |
|  | Mouse 3 |  |  |  | Mouse 3 | 3.66 | 1.89 |
|  | Mouse 4 |  |  |  | Mouse 4 |  |  |
|  | Mouse 5 |  |  |  | Mouse 5 | 3.82 | 2.92 |
|  | Mouse 6 |  |  |  | Mouse 6 | 4.13 | 3.06 |
|  | Mouse 7 |  |  |  | Mouse 7 | 3.72 | 3.3 |
|  | Mouse 8 |  |  |  | Mouse 8 | 4.73 | 3.53 |
|  | Mouse 9 |  |  |  | Mouse 9 | 3.68 | 3.54 |
|  | Mouse 10 |  |  |  | Mouse 10 | 4.52 | 3.28 |
|  | Mouse 11 |  |  |  | Mouse 11 |  |  |
|  | Mouse 12 |  |  |  | Mouse 12 | 2.56 | 2.17 |
|  | Mouse 13 |  |  |  | Mouse 13 | 4.83 | 2.28 |
|  | Mouse 14 |  |  |  | Mouse 14 | 3 | 2.84 |
|  | Mouse 15 |  |  |  | Mouse 15 | 3.59 | 2.05 |
|  | Mouse 16 |  |  |  | Mouse 16 |  |  |
|  | Mouse 17 |  |  |  | Mouse 17 | 3.68 | 2.68 |
|  | Mouse 18 |  |  |  | Mouse 18 | 4.08 | 3.13 |
|  | Mouse 19 |  |  |  | Mouse 19 | 2.42 | 2.16 |
|  | Mouse 20 |  |  |  | Mouse 20 | 4.26 | 2.85 |
|  | Mouse 21 |  |  |  | Mouse 21 | 3.5 | 2.15 |
|  | Mouse 22 |  |  |  | Mouse 22 | 3.27 | 2.91 |
|  | Mouse 23 |  |  |  | Mouse 23 | 5.6 | 2.62 |
|  | Mouse 24 |  |  |  | Mouse 24 |  |  |
| Week 4 |  | Lymphedema | Control | Week 8 |  | Lymphedema | Control |
|  | Mouse 1 | 3.04 | 2.83 |  | Mouse 1 | 2.95 | 2.72 |
|  | Mouse 2 |  |  |  | Mouse 2 |  |  |
|  | Mouse 3 | 2.39 | 2.27 |  | Mouse 3 | 2.73 | 2.21 |
|  | Mouse 4 |  |  |  | Mouse 4 |  |  |
|  | Mouse 5 | 3 | 1.49 |  | Mouse 5 | 2.6 | 2.59 |
|  | Mouse 6 | 4.27 | 3.11 |  | Mouse 6 | 3.92 | 1.84 |
|  | Mouse 7 | 2.97 | 2.39 |  | Mouse 7 | 2.96 | 2.22 |
|  | Mouse 8 | 3.15 | 1.73 |  | Mouse 8 | 2.96 | 2 |
|  | Mouse 9 | 3.06 | 2.75 |  | Mouse 9 | 2.67 | 2.96 |
|  | Mouse 10 | 3.31 | 2.1 |  | Mouse 10 | 2.73 | 2.7 |
|  | Mouse 11 |  |  |  | Mouse 11 |  |  |
|  | Mouse 12 | 2.16 | 2.4 |  | Mouse 12 | 2.8 | 2.32 |
|  | Mouse 13 | 3.93 | 2.69 |  | Mouse 13 | 2.45 | 2.2 |
|  | Mouse 14 | 2.91 | 1.69 |  | Mouse 14 | 2.75 | 2.56 |
|  | Mouse 15 | 3.08 | 2.67 |  | Mouse 15 | 3.01 | 2.93 |
|  | Mouse 16 |  |  |  | Mouse 16 |  |  |
|  | Mouse 17 | 2.97 | 1.61 |  | Mouse 17 | 2.82 | 2.58 |
|  | Mouse 18 | 3.11 | 2.56 |  | Mouse 18 | 2.23 | 2.56 |
|  | Mouse 19 | 2 | 1.83 |  | Mouse 19 | 2.7 | 1.5 |
|  | Mouse 20 | 2.16 | 2.05 |  | Mouse 20 | 2.66 | 2.32 |
|  | Mouse 21 | 3.77 | 2.81 |  | Mouse 21 | 2.38 | 1.99 |
|  | Mouse 22 | 2.65 | 1.9 |  | Mouse 22 | 2.66 | 2.32 |
|  | Mouse 23 | 4.82 | 1.7 |  | Mouse 23 | 3.41 | 2.7 |
|  | Mouse 24 |  |  |  | Mouse 24 |  |  |

**Supplemental figure and table legends**

Supplemental figure 1 legend: Scatter plot between μ-CT scans and electronic caliper

Blue dots = rater 1 (AB). red dots = rater 2 (AW). green dots = rater 3 (FD). CT: computed tomography. μ=micro

Supplemental figure 2 legend: Scatter plot between μ-CT scans and plethysmometer

Blue dots = rater 1 (AB). red dots = rater 2 (AW). green dots = rater 3 (FD). CT: computed tomography. μ=micro

Supplemental table 1 legend

μ-CT-measurements conducted by rater 1 (AB) in week 1. 2. 4 and 8. Four mice were euthanized due to ethical concerns. Mouse number 4 died during anesthesia while being μ-CT-scanned. Measurements of mouse number 19 in week 8 are unavailable as we had technical problems with the μ-CT-scanner. Volume is in mm^3^

CT: computed tomography. μ=micro. lymphedema = right hindlimb. control = left hindlimb. mm^3^ = cubic millimeters

Supplemental table 2 legend

Plethysmometer measurements conducted by rater 1 (AB) in week 2. 4 and 8. Week 1 measurements are unavailable due to technical issues. see Discussion. Four mice were euthanized due to ethical concerns. Mouse number 4 died during anesthesia while being μ-CT-scanned. Volume is in ml.

Lymphedema = right hindlimb. control = left hindlimb. ml = milliliter

Supplemental table 3 legend

Electronic caliper measurements conducted by rater 1 (AB) in week 2. 4 and 8. Week 1 measurements are unavailable due to technical issues. see Discussion. Four mice were euthanized due to ethical concerns. Mouse number 4 died during anesthesia while being μ-CT-scanned. Volume is in mm.

Lymphedema = right hindlimb. control = left hindlimb. mm = millimeters.

Supplemental table 4 legend

μ-CT-measurements conducted by rater 2 (AW) in week 1. 2. 4 and 8. Four mice were euthanized due to ethical concerns. Mouse number 4 died during anesthesia while being μ-CT-scanned. Measurements of mouse number 19 in week 8 are unavailable as we had technical problems with the μ-CT-scanner. Volume is in mm^3^

CT: computed tomography. μ=micro. lymphedema = right hindlimb. control = left hindlimb. mm^3^ = cubic millimeters

Supplemental table 5 legend

Plethysmometer measurements conducted by rater 2 (AW) in week 2. 4 and 8. Week 1 measurements are unavailable due to technical issues. see Discussion. Four mice were euthanized due to ethical concerns. Mouse number 4 died during anesthesia while being μ-CT-scanned. Volume is in ml.

Lymphedema = right hindlimb. control = left hindlimb. ml = milliliter

Supplemental table 6 legend

Electronic caliper measurements conducted by rater 2 (AW) in week 2. 4 and 8. Week 1 measurements are unavailable due to technical issues. see Discussion. Four mice were euthanized due to ethical concerns. Mouse number 4 died during anesthesia while being μ-CT-scanned. Volume is in mm.

Lymphedema = right hindlimb. control = left hindlimb. mm = millimeters.

Supplemental table 7 legend

μ-CT-measurements conducted by rater 3 (FD) in week 1. 2. 4 and 8. Four mice were euthanized due to ethical concerns. Mouse number 4 died during anesthesia while being μ-CT-scanned. Measurements of mouse number 19 in week 8 are unavailable as we had technical problems with the μ-CT-scanner. Volume is in mm^3^

CT: computed tomography. μ=micro. lymphedema = right hindlimb. control = left hindlimb. mm^3^ = cubic millimeters

Supplemental table 8 legend

Plethysmometer measurements conducted by rater 3 (FD) in week 2. 4 and 8. Week 1 measurements are unavailable due to technical issues. see Discussion. Four mice were euthanized due to ethical concerns. Mouse number 4 died during anesthesia while being μ-CT-scanned. Volume is in ml.

Lymphedema = right hindlimb. control = left hindlimb. ml = milliliter

Supplemental table 9 legend

Electronic caliper measurements conducted by rater 3 (FD) in week 2. 4 and 8. Week 1 measurements are unavailable due to technical issues. see Discussion. Four mice were euthanized due to ethical concerns. Mouse number 4 died during anesthesia while being μ-CT-scanned. Volume is in mm.

Lymphedema = right hindlimb. control = left hindlimb. mm = millimeters.
